# Genome-wide association study identifies 14 previously unreported susceptibility loci for adolescent idiopathic scoliosis in Japanese

**DOI:** 10.1038/s41467-019-11596-w

**Published:** 2019-08-15

**Authors:** Ikuyo Kou, Nao Otomo, Kazuki Takeda, Yukihide Momozawa, Hsing-Fang Lu, Michiaki Kubo, Yoichiro Kamatani, Yoji Ogura, Yohei Takahashi, Masahiro Nakajima, Shohei Minami, Koki Uno, Noriaki Kawakami, Manabu Ito, Ikuho Yonezawa, Kei Watanabe, Takashi Kaito, Haruhisa Yanagida, Hiroshi Taneichi, Katsumi Harimaya, Yuki Taniguchi, Hideki Shigematsu, Takahiro Iida, Satoru Demura, Ryo Sugawara, Nobuyuki Fujita, Mitsuru Yagi, Eijiro Okada, Naobumi Hosogane, Katsuki Kono, Masaya Nakamura, Kazuhiro Chiba, Toshiaki Kotani, Tsuyoshi Sakuma, Tsutomu Akazawa, Teppei Suzuki, Kotaro Nishida, Kenichiro Kakutani, Taichi Tsuji, Hideki Sudo, Akira Iwata, Tatsuya Sato, Satoshi Inami, Morio Matsumoto, Chikashi Terao, Kota Watanabe, Shiro Ikegawa

**Affiliations:** 1 Laboratory for Bone and Joint Diseases, Center for Integrative Medical Sciences, RIKEN, Tokyo, 108-8639 Japan; 2 Department of Orthopedic Surgery, Keio University School of Medicine, Tokyo, 160-8582 Japan; 3 Laboratory for Genotyping Development, Center for Integrative Medical Sciences, RIKEN, Yokohama, 230-0045 Japan; 4 School of Pharmacy, Taipei Medical University, Taipei, 11042 Taiwan; 5 Laboratory for Statistical Analysis, Center for Integrative Medical Sciences, RIKEN, Yokohama, 230-0045 Japan; 6 Kyoto-McGill International Collaborative School in Genomic Medicine, Kyoto University Graduate School of Medicine, Kyoto, 606-8501 Japan; 7 Department of Orthopedic Surgery, Seirei Sakura Citizen Hospital, Sakura, 285-8765 Japan; 8 Department of Orthopedic Surgery, National Hospital Organization, Kobe Medical Center, Kobe, 654-0155 Japan; 9 Department of Orthopedic Surgery, Meijo Hospital, Nagoya, 460-0001 Japan; 10 Department of Orthopedic Surgery, National Hospital Organization, Hokkaido Medical Center, Sapporo, 063-0005 Japan; 11 Department of Orthopedic Surgery, Juntendo University School of Medicine, Tokyo, 113-8421 Japan; 12 Department of Orthopedic Surgery, Niigata University Medical and Dental General Hospital, Niigata, 951-8510 Japan; 13 Department of Orthopedic Surgery, Osaka University Graduate School of Medicine, Suita, 565-0871 Japan; 14 Department of Orthopedic Surgery, Fukuoka Children’s Hospital, Fukuoka, 813-0017 Japan; 15 Department of Orthopedic Surgery, Dokkyo Medical University School of Medicine, Mibu, 321-0293 Japan; 16 Department of Orthopedic Surgery, Kyushu University Beppu Hospital, Beppu, 874-0838 Japan; 17 Department of Orthopedic Surgery, Faculty of Medicine, The University of Tokyo, Tokyo, 113-8655 Japan; 18 Department of Orthopedic Surgery, Nara Medical University, Kashihara, 634-8522 Japan; 19 Department of Orthopedic Surgery, Dokkyo Medical University Koshigaya Hospital, Koshigaya, 343-8555 Japan; 20 Department of Orthopedic Surgery, Kanazawa University Hospital, Kanazawa, 920-8641 Japan; 21 Department of Orthopedic Surgery, Jichi Medical University, Shimotsuke, 329-0468 Japan; 22 Department of Orthopedic Surgery, Saiseikai Central Hospital, Tokyo, 108-0073 Japan; 23 Department of Orthopedic Surgery, National Defense Medical College, Tokorozawa, 359-8513 Japan; 24 Department of Orthopedic Surgery, Eiju General Hospital, Tokyo, 110-8645 Japan; 25 Department of Orthopedic Surgery, Kobe University Graduate School of Medicine, Kobe, 650-0017 Japan; 26 Department of Advanced Medicine for Spine and Spinal Cord Disorders, Hokkaido University Graduate School of Medicine, Sapporo, 060-8638 Japan; 27 Department of Preventive and Therapeutic Research for Metastatic Bone Tumor, Faculty of Medicine and Graduate School of Medicine, Hokkaido University, Sapporo, 060-8638 Japan

**Keywords:** Bone, Disease genetics, Genome-wide association studies

## Abstract

Adolescent idiopathic scoliosis (AIS) is the most common pediatric spinal deformity. Several AIS susceptibility loci have been identified; however, they could explain only a small proportion of AIS heritability. To identify additional AIS susceptibility loci, we conduct a meta-analysis of the three genome-wide association studies consisting of 79,211 Japanese individuals. We identify 20 loci significantly associated with AIS, including 14 previously not reported loci. These loci explain 4.6% of the phenotypic variance of AIS. We find 21 *cis*-expression quantitative trait loci-associated genes in seven of the fourteen loci. By a female meta-analysis, we identify additional three significant loci. We also find significant genetic correlations of AIS with body mass index and uric acid. The cell-type specificity analyses show the significant heritability enrichment for AIS in multiple cell-type groups, suggesting the heterogeneity of etiology and pathogenesis of AIS. Our findings provide insights into etiology and pathogenesis of AIS.

## Abstract (web summary)

Adolescent idiopathic scoliosis (AIS) is a common pediatric disease leading to spinal deformities. Here, the authors report GWAS followed by genome-wide meta-analysis in up to 79,211 Japanese individuals, identifying 20 genetic loci for AIS, 14 of which were previously unreported, and perform in vitro validation for rs1978060.

## Introduction

AIS is a complex spinal deformity which is defined as a lateral spinal curvature with a Cobb angle of >10 degrees^1^. AIS is a common disease affecting ~2.5% of adolescents in Japan^2^ and millions of children worldwide are affected with a prevalence of 2–3%^3,4^. AIS is regarded as a multifactorial disease affected by genetic and environmental factor^5^. The importance of genetic factors in the etiology and pathogenesis of AIS has been demonstrated by many twin, family and population studies^6,7^.

Genome-wide association study (GWAS) is one of the most effective methods to identify genetic factors of complex traits, including common diseases. To date, several GWASs have been conducted for AIS^8–13^. We previously conducted two GWASs (GWAS1 and GWAS2) in Japanese populations and identified three loci significantly associated with AIS susceptibility (10q24.31, 6q24.1 and 9p22.2)^8–10^. A subsequent meta-analysis using multi-ethnic cohorts confirmed their robust associations^14^. A total of seven significantly associated loci have been identified in the meta-analysis; however, the proportion of the AIS heritability explained by the seven loci is estimated to be only ~3%. Therefore, it is indispensable to identify additional susceptibility loci for understanding the etiology and pathogenesis of AIS.

In the present study, we perform a large Japanese GWAS followed by a meta-analysis of three GWASs. We replicate the association of 6 previous reported susceptibility loci and identify 14 previously unreported susceptibility loci. By a sex-stratified analysis, we further identify three significant loci for female AIS. The integrative analyses indicate the significant genetic correlations of AIS with body mass index (BMI) and uric acid (UA), and show the significant heritability enrichment for AIS in multiple cell-type groups. In addition, we find 21 *cis*-expression quantitative trait loci (eQTL)-associated genes in 7 out of 14 previously unreported susceptibility loci. In vitro functional analyses suggest that one of these eQTLs, rs1978060, regulates the expression of *TBX1*, and the difference in FOXA2 binding causes difference in *cis-*acting transcriptional regulatory function between alleles. These findings provide insights into etiology and pathogenesis of AIS.

## Results

### Association analysis

**Fig. 1:**
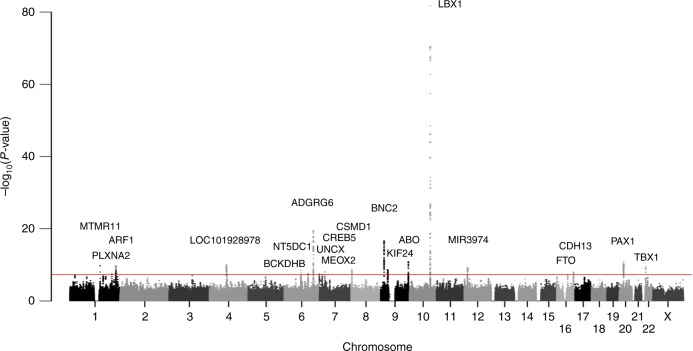
Manhattan plot showing the –log_10_ *P* value for the SNPs in the AIS GWAS. *P* values were derived from the meta-analysis by using the inverse-variance method under a fixed-effect model. The red line represents the genome-wide significance threshold (*P* = 5 × 10^−8^). The genetic loci that satisfied the genome-wide significance threshold in the meta-analysis of the three GWAS are labeled

**Fig. 2:**
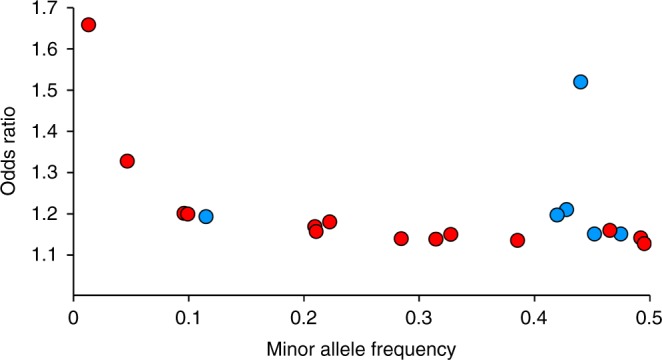
Relationship between effect size and minor allele frequency. The meta-analysis effect size (*y* axis) and the minor allele frequency (*x* axis) for 20 significant SNPs. Red circles represent the SNPs at previously unreported loci (*n* = 14) and blue circles represent the SNPs at previously reported loci (*n* = 6). Effect sizes are measured as odds ratios, which give the odds of the outcome given exposure to one risk allele compared with those to no risk allele

To identify the additional AIS susceptibility loci, we conducted a large GWAS (GWAS3: 3254 cases and 63,252 controls). In addition, we reanalyzed the previous GWASs (GWAS1 and GWAS2) by updating the reference panel (Methods). For each of the three GWASs, imputation analysis was performed separately. Subsequently, the meta-analysis combining the three GWASs was performed (a total of 5327 cases and 73,884 controls; Supplementary Fig. 1). The genomic control inflation factor (*λ*_GC_ = 1.16) showed an inflation in the GWAS; however, the linkage disequilibrium (LD) score regression analysis indicated that the inflation was mostly from polygenicity (85.5%) and biases have a small contribution (estimated mean *χ*^2^ = 1.29 and LD score intercept = 1.04). Compared to the previously reported GWAS, the bias was not large^15^; we therefore did not apply the GC correction. As a result of the meta-analysis, we identified 20 significant AIS loci including 14 previously unreported loci (Table 1, Supplementary Data 1, Fig. 1 and Supplementary Fig. 2). The most significant association was identified at rs11190870 (*P* *=* 2.01 × 10^−82^) as with our previous GWASs^8,10^. Including this locus, significant associations of the six previously reported loci (10q24.31, 6q24.1, 9p22.2, 20p11.22, 16q23.3 and 9q34.2)^14^ were replicated in the present meta-analysis (Table 1, Supplementary Data 1 and Fig. 1). Lead variants at the 14 previously unreported loci included both common genetic variants with smaller effect size and low-frequency genetic variants with relatively large effects sizes (Fig. 2 and Supplementary Table 1). Of the 14 variants, 3 variants had distinct minor allele frequency (MAF) spectra between Japanese (JPN) and Europeans (EUR). These three variants are rare (MAF < 0.01) or monomorphic in EUR, but low-frequency (MAF: 0.01–0.05) or common (MAF > 0.05) in JPN (Fig. 2 and Supplementary Table 1). We estimated the AIS heritability using LD score regression. The SNP heritability estimated on the liability scale by the LDSC software (https://github.com/bulik/ldsc) with the use of common variants in hapmap3 data is 42%. The lead variants of the 20 significant loci explained 4.6% of the phenotypic variance. We performed a stepwise conditional analysis to detect multiple independent signals within the 20 significant loci. We found five additional signals that reached the locus-wide significance (*P* < 5.0 × 10^−6^) (Supplementary Table 2). These five signals additionally explained 0.6% of the phenotypic variance (Supplementary Table 2 and Supplementary Fig. 3). These results were also confirmed by GCTA-COJO (Supplementary Table 3 and Supplementary Note 1).

**Table 1:** Association of the genome-wide significant loci

SNP	Chr.	Pos.	Gene in or near Region of association	RA	RAF Case	RAF Control	*P* value^a^	OR	95% CI	*P*_het_	Genotyped/imputation	Min Rsq
Previously unreported loci												
rs141903557	4q21.23	85168056	LOC101928978	C	0.060	0.047	9.78 × 10^−11^	1.33	1.22-1.45	0.52	Imputed	0.98
rs11205303	1q21.2	149906413	MTMR11	C	0.24	0.21	1.62 × 10^−10^	1.17	1.11-1.23	0.91	Genotyped	0.94
rs12029076	1q42.13	228272687	ARF1	G	0.81	0.78	2.17 × 10^−10^	1.18	1.12-1.24	0.40	Imputed	0.99
rs1978060	22q11.21	19749525	TBX1	G	0.49	0.47	3.26 × 10^−10^	1.16	1.11-1.22	0.62	Imputed	0.72
rs2467146	12p12.3	17800607	LINC02378/MIR3974	A	0.70	0.67	5.96 × 10^−10^	1.15	1.10-1.20	0.23	Imputed	0.99
rs11787412	8p23.2	3134239	CSMD1	A	0.42	0.38	1.32 × 10^−9^	1.14	1.09-1.18	0.86	Imputed	0.99
rs188915802	9p13.3	34318683	KIF24	T	0.019	0.013	1.94 × 10^−9^	1.66	1.41-1.96	0.05	Imputed	0.81
rs658839	6q14.1	81228722	BCKDHB/FAM46A	G	0.54	0.51	3.15 × 10^−9^	1.14	1.09-1.19	0.88	Imputed	0.85
rs160335	7p15.1	28587817	CREB5	G	0.54	0.51	9.10 × 10^−9^	1.13	1.08-1.18	0.69	Imputed	0.96
rs482012	6q22.1	116430533	NT5DC1	T	0.74	0.72	2.30 × 10^−8^	1.14	1.09-1.19	0.15	Genotyped	0.99
rs11341092	7p22.3	1269592	LOC101927021/UNCX	AC	0.33	0.31	2.92 × 10^−8^	1.14	1.09-1.19	0.93	Imputed	0.81
rs17011903	1q32.2	208259531	PLXNA2	A	0.11	0.10	3.56 × 10^−8^	1.20	1.13-1.28	0.11	Imputed	0.98
rs397948882	7p21.2	15636869	AGMO/MEOX2	A	0.11	0.10	3.66 × 10^−8^	1.20	1.12-1.28	0.72	Imputed	0.98
rs12149832	16q12.2	53842908	FTO	G	0.82	0.79	4.40 × 10^−8^	1.16	1.10-1.22	0.34	Genotyped	0.96
Previously reported loci												
rs11190870	10q24.31	102979207	LINC01514/LBX1	T	0.66	0.56	2.01 × 10^−82^	1.52	1.46-1.59	0.22	Genotyped	1
rs9389985	6q24.1	142653898	ADGRG6	G	0.48	0.43	3.51 × 10^−20^	1.21	1.16-1.26	0.43	Imputed	0.96
rs7028900	9p22.2	16690612	BNC2	C	0.46	0.42	2.19 × 10^−17^	1.20	1.15-1.25	0.93	Imputed	0.93
rs144131194	9q34.2	136145993	ABO	AAGAAGGGAAATTAATAAATATT	0.58	0.55	1.35 × 10^−11^	1.15	1.11-1.20	0.96	Imputed	1
rs6047716	20p11.22	21894005	PAX1/LINC01432	C	0.51	0.47	1.45 × 10^−11^	1.15	1.11-1.20	0.77	Imputed	0.97
rs2194285	16q23.3	82894817	CDH13	G	0.13	0.11	8.69 × 10^−9^	1.19	1.12-1.27	0.80	Genotyped	1

*Chr.* chromosome, *Pos.* genomic position (GRCh37/hg19), *RA* risk allele, *RAF* risk allele frequency, *OR* odds ratio, *CI* confidence interval, *P*_het_ *P* values for heterogeneity from Cochran’s *Q*-test

^a^The combined *P* values were calculated by the inverse-variance method under a fixed-effect model

### Integrative analysis

AIS is a complex disease and several hypotheses including neuromuscular, biomechanical, genetic, developmental and growth-related have been proposed to explain the etiology and pathogenesis of AIS^5,16^. However, there is currently no hypothesis commonly supported. Therefore, the identification of AIS-related tissues and cell types is valuable for understanding of etiology and pathogenesis of AIS. We investigated the cell-type specificity of AIS based on the enrichment of heritability. We applied stratified LD score regression to our GWAS result using 220 cell-type-specific annotations of the four histone marks (H3K4me1, H3K4me3, H3K9ac and H3K27ac) constructed by the Roadmap Epigenomics Project^17^. First, to obtain an overview of AIS-related cell types, we assessed heritability enrichment of 10 major cell-type groups that are constructed reflecting system- or organ-level structures from the 220 individual cell-type-specific annotations^18^. The most significant enrichment was observed in the cardiovascular cell group (*P* = 1.07 × 10^−5^) (Supplementary Table 4). In addition, significant enrichment was also observed in other five cell-type groups: skeletal muscle, connective or bone, other, central nervous system (CNS) and gastrointestinal groups (*P* < 5.0 × 10^−3^) (Supplementary Fig. 4 and Supplementary Table 4). We further assessed the heritability enrichment of the 220 individual cell types and identified significant enrichments in six cell types (*P* < 2.3 × 10^−4^) (Supplementary Data 2). The most significant heritability enrichment was observed in H3K4me1 in fetal stomach cell (*P* = 4.82 × 10^−6^) and significant enrichment was also observed in H3K4me1 in other fetal organs such as lung, trunk muscle and leg muscle. Significant enrichment was also observed in H3K4me1 in stomach smooth muscle and H3K4me3 in penile foreskin fibroblast primary. These results suggest that the etiology and pathogenesis of AIS are heterogeneous and multifactorial.

To gain biological insights, we conducted a pathway analysis with the result of the meta-analysis using PASCAL^19^ (https://www2.unil.ch/cbg/index.php?title=Pascal). Sixty-two pathways showed the nominal significance (*P* < 0.05); however, no specific pathway significantly associated with AIS (*P* < 4.6 × 10^−5^) was detected (Supplementary Data 3). To obtain insights into the genetic architecture of AIS, we explored the shared genetics between AIS and various traits. We calculated genetic correlation between this study and 67 complex human traits (61 quantitative traits and 6 diseases) in Japanese^20,21^ using bivariate LD score regression^22^ (Supplementary Data 4). Genetic correlations of AIS with BMI and UA have been suggested in our previous studies^20,21^ and their significant negative correlations were replicated in this study (BMI: *r*_g_ = −0.15, *P* = 3.14 × 10^−5^; UA: *r*_g_ = −0.15, *P* = 1.22 × 10^−5^).

### Sex-stratified analysis

Since AIS has a clear sex difference of female predominance^23^ and the number of male case samples was limited, we conducted a female-specific meta-analysis of the GWASs (5004 cases and 33,679 controls). As a result, 15 loci showed the genome-wide significance (Supplementary Data 5). Three of the fifteen loci (3q13.2, 1q25.2 and 1q23.3) did not reach GWAS significance in the overall study. We found an evidence of sex-heterogeneity in the *BOC* region on chromosome 3 (Table 2).

**Table 2:** Association of the genome-wide significant loci for female AIS (only loci FEMALE-sig)

SNP	Chr.	Pos.	Gene in or near region of association	RA	F_RAF Case	F_RAF Control	F_OR (95% CI)	F_*P* value^a^	M_RAF Case	M_RAF Control	M_OR (95% CI)	M_*P* value^a^	*P*_het_
rs73235136	3q13.2	112951529	BOC	C	0.50	0.46	1.15 (1.10–1.20)	3.45 × 10^−9^	0.44	0.47	0.88 (0.75–1.03)	1.01 × 10^−1^	0.00057
rs545608	1q25.2	177899121	SEC16B	G	0.76	0.73	1.16 (1.10–1.23)	1.03 × 10^−8^	0.75	0.74	1.05 (0.88–1.25)	6.15 × 10^−1^	0.13
rs142502288	1q23.3	162450931	SH2D1B/UHMK1	G	0.028	0.020	1.52 (1.31–1.76)	3.11 × 10^−8^	0.025	0.023	1.10 (0.66–1.84)	7.13 × 10^−1^	0.88

*Chr.* chromosome, *Pos.* genomic position (GRCh37/hg19), *F* female, *M* male, *RA* risk allele, *RAF* risk allele frequency, *OR* odds ratio, *CI* confidence interval, *P*_het_ *P* values for heterogeneity from Cochran’s *Q*-test

^a^The combined *P* values were calculated by the inverse-variance method under a fixed-effect model

### Functional annotation and candidate susceptibility genes

To identify candidate causal variants at each of the 14 previously unreported loci (Supplementary Fig. 2), we used several SNP annotation tools (HaploReg (https://pubs.broadinstitute.org/mammals/haploreg/haploreg.php), 3DSNP (http://cbportal.org/3dsnp/), RegulomeDB (http://regulomedb.org/), etc.) and explored the biological role of these variants. For each locus, we searched all variants in high LD (*r*^2^ > 0.8 in East Asians (EAS) of 1KGP3) with the most associated variant (lead variant) and found some candidate variants with regulatory function (Supplementary Data 6). Furthermore, to identify susceptibility genes in the loci, we searched for eQTL using data from the Genotype-Tissue Expression (GTEx) project^24^ (http://www.gtexportal.org/home/). As demonstrated by the cell-type-specific analysis, AIS is a multifactorial heterogeneous disease and multiple tissues may be associated with AIS. Moreover, because the tissues used in the GTEx project are limited and there are no data on AIS-related tissues such as intervertebral disc, cartilage and bone, we searched data on all tissues currently available. We observed significant *cis*-eQTLs at 7 of the 14 previously unreported loci, and the expression levels of 21 genes in the 7 *cis*-eQTL loci were associated with some variants that were in high LD (*r*^2^ > 0.8 in EAS) with the lead variants (Supplementary Table 5). We also searched for candidate variants with regulatory functions and eQTL using GTEx data for three previously unreported loci that were significant in the female meta-analysis (Supplementary Data 7 and Supplementary Table 6). The expression levels of three genes in the chromosome 3 locus were associated with some variants that were in high LD (*r*^2^ > 0.8 in EAS) with the lead variants (Supplementary Table 6). These *cis*-eQTL-associated genes are promising candidates for AIS susceptibility genes, among which there are several interesting candidate genes (Supplementary Table 7).

Among these *cis*-eQTLs, we specifically focused on rs1978060, the lead variant at chromosome 22q11.21 corresponding to the lead *cis*-eQTL variant of *TBX1*. *TBX1* is a member of the T-box gene family, which is a group of transcription factors involved in the regulation of developmental processes. Mutations of *TBX1* are known to cause DiGeorge syndrome (OMIM #188400) and velocardiofacial syndrome (OMIM #192430), and scoliosis is one of the clinical features, which is highly prevalent (47–49%) in association with these syndromes^25–28^. There is substantial evidence that *Tbx1* haploinsufficiency is responsible for the physical features of these syndromes, and it has also been reported that *Tbx1* knockout mice show vertebral anomalies^29,30^. In addition, other T-box gene family member, *TBX6* is known to cause congenital scoliosis^31^. We therefore selected the *TBX1* locus for further functional analysis. To prioritize candidate variants at the locus, we used a simple scoring system. The candidate variants were scored when they possess the following functional information: (i) promoter histone mark, (ii) enhancer histone mark, (iii) DNase protein binding and (iv) motif change. Based on the system, we selected rs1978060 as a most likely causal variant at this locus. rs1978060 was also a promising candidate causal SNP in other scoring systems such as 3DSNP and RegulomeDB (Supplementary Data 6).

**Fig. 3:**
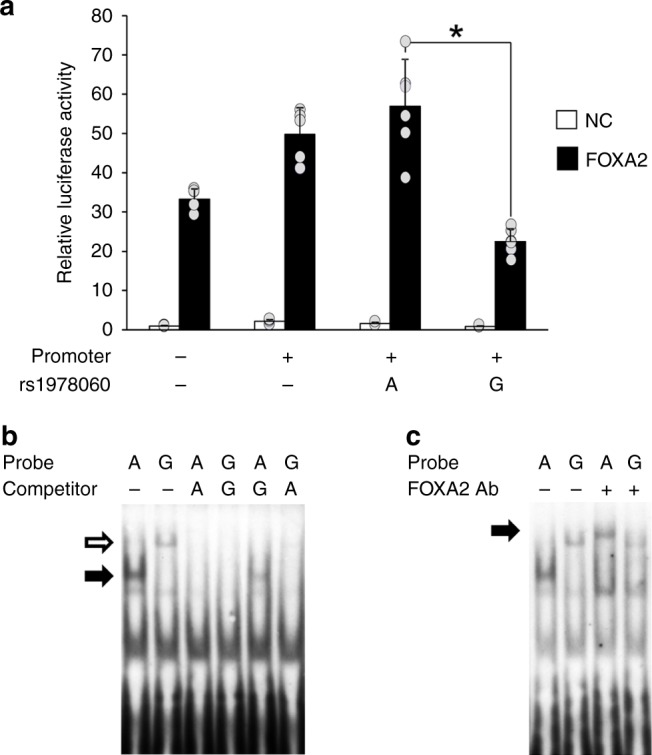
Allelic difference of functional variant, rs1978060 in Chr.22q11.21. **a** Reporter assays in MCF-7 cells. There was a significantly decreased transcriptional activity for the risk G-allele of rs1978060 compared to the non-risk A-allele. Error bars show standard deviation (S.D.) for each variant. Asterisks indicate statistically significant changes in paired comparison (*t*-test *P* < 0.01). *n* = 2 independent experiments. **b** Electrophoretic mobility shift assays with nuclear extracts from MCF-7 cells. There was specific bands for A-allele probe (lane 1, black arrow) and the G-allele probe (lane 2, white arrow) of rs1978060. Competition analyses were performed using an excess of the unlabeled A-allele probe (lane 3, lane 6) and G-allele probe (lane 4, lane 5) as competitors. **c** A super-shift assay using the FOXA2 antibody. The black arrow indicates a super-shifted FOXA2 complex in lane 3. Source data are provided as a Source Data file

We conducted in vitro analysis for rs1978060. We constructed luciferase reporter vectors by cloning the *TBX1* promoter region and inserting oligonucleotides that contained either the risk or non-risk allele of rs1978060. We evaluated the effect of rs1978060 on *TBX1* promoter activity and revealed that the risk allele-G significantly reduced the reporter activity compared to the non-risk allele-A (Fig. 3a and Supplementary Fig. 5a). We then performed an electrophoretic mobility shift assay to examine the DNA–protein binding of rs1978060. As expected, different binding patterns of DNA–protein complexes were observed between the risk and non-risk alleles of rs1978060 (Fig. 3b and Supplementary Fig. 5b). We searched for possible transcription factors that have the differential binding effect on rs1978060 using annotation tools. FOXA2 was predicted by JASPER (http://jaspar.genereg.net) to have a higher binding score in the non-risk allele and binding to this genomic region has also been demonstrated by chromatin immunoprecipitation assays. Furthermore, an electrophoretic mobility shift assay using FOXA2 antibody showed a super-shift in the presence of the antibody (Fig. 3c and Supplementary Fig. 5b) and the effect of rs1978060 on the *TBX1* promoter activity showed significant allelic difference when co-transfection with FOXA2 (Fig. 3a and Supplementary Fig. 5a). These results indicate that FOXA2 binds to rs1978060 and regulates the transcription of *TBX1*.

## Discussion

In the present study, we conducted a large AIS GWAS in Japanese and performed a meta-analysis with two previous Japanese GWASs. With larger sample sizes and improved imputation, we identified 20 susceptibility loci including 14 previously unreported loci. The larger sample size yielded both common variants with smaller effect sizes and low-frequency variants with relatively large effects on the risk of AIS, suggesting contribution of both rare and common variants in the genetic architecture of AIS (Fig. 2). Of the lead variants at the 14 previously unreported loci, 11 were common variants (MAF > 0.05) in both JPN and EUR. Some of these common variants differed greatly in MAF between JPN and EUR (Supplementary Table 1). However, the association of common lead variants (MAF > 0.05) at the three susceptibility loci (10q24.31, 6q24.1 and 9p22.2) identified in our previous GWAS was replicated even if there is a large difference in MAF between JPN and EUR^14,32,33^. Therefore, it is expected that the association of common lead variants (MAF > 0.05) identified in the present study will also be replicated in a multi-ethnic meta-analysis. From this point of view, rs73235136, a common lead variant (MAF > 0.05) at a female-specific susceptibility locus, also has a large difference in MAF between JPN and EUR, but the association is expected to be replicated. On the other hand, the low-frequency variants (rs141903557 and rs188915802), which have relatively large effects, are rare or monomorphic in EUR (Supplementary Table 1). The contribution of these variants with relatively large effects to the genetic architecture of AIS can differ among ethnic groups, as no association can be found unless there are other causal variants.

Besides our GWASs, a few GWASs in Caucasian and in Chinese have been reported^11–13^. The association of 20p11.22 locus reported in Caucasian GWAS was replicated in this study; however, the association of other loci reported in Chinese GWAS was not replicated. These loci were also not significant in our previous multi-ethnic meta-analysis^14^. They seem to be Chinese population-specific signals. Recently, an exome-wide association study identified that rs13107325, a non-synonymous SNP encoding SLC39A8, is associated with AIS^34^. However, the SNP is monomorphic in Japanese and other East Asian populations, and no variants associated with AIS at this locus were identified.

Integrative analyses indicated that AIS is associated with several cell-type groups such as cardiovascular, connective bone, skeletal muscle, and CNS, which suggests heterogeneity in etiology and pathogenesis of AIS. The negative correlation between AIS and BMI confirmed by our genetic analysis is consistent with the clinical observations reported in many clinical studies^35,36^. In contrast, there have been few reports on the relation between AIS and UA. We could find only a few papers reporting a relation between renal and ureteral abnormalities and congenital scoliosis^37,38^. UA is also known to be associated with a risk for cardiovascular diseases, and our cell-type specificity analysis showed that AIS is associated with cardiovascular cell types. These findings provide starting points to clarify the complex etiology and pathogenesis of AIS.

In the *cis*-eQTL analysis, we searched data for all tissues included in the current GTEx project; however, the tissues examined in the project are limited. AIS-related tissues such as intervertebral disc, cartilage and bone were not included and in the first place, it is difficult to determine the tissue that contributes to the onset of AIS. In addition, because the sample size greatly affects eQTL mapping, current eQTL data would have missed a considerable number of actually associated tissues. However, based on the *cis-*eQTL analysis followed by functional annotations of the variants, we could show that the *TBX1* expression is regulated through the binding of FOXA2 to rs1978060. Consistent with our results, previous mouse studies have shown that Foxa2 in the pharyngeal endoderm can bind and activate transcription through the critical *cis*-element upstream of *Tbx1* (refs. ^39,40^).

Among the *cis*-eQTL-associated genes other than *TBX1*, there are several interesting candidate genes. One of them is *DSE* at chromosome 6q22.1. *DSE* encodes dermatan sulfate epimerase, an enzyme that is necessary for dermatan sulfate biosynthesis. Bi-allelic loss-of-function mutations in *DSE* are reported to cause Ehlers–Danlos syndrome (EDS) musculocontractural type 2 (OMIM #615539), which is characterized by progressive multi-system fragility-related manifestations. The patients of this type of EDS present spinal deformity including scoliosis and kyphoscoliosis^36^. Consistent with this, *cis*-eQTL analysis shows that risk allele of rs482012 is associated with decreased expression of *DSE* (Supplementary Table 5). In addition, *FTO* on chromosome 16q12.2 is a promising candidate susceptibility gene. AIS-associated variants including the lead SNP, rs12149832 are present in the intronic region of *FTO* and the risk allele is associated with decreased expression of *FTO* (Supplementary Table 5 and Supplementary Data 6). It is well known that *FTO* is associated with BMI and obesity^41–43^, and risk allele of rs12149832 is associated with lower BMI and decreased risk of obesity^43^. These findings are consistent with the recently reported negative correlation between BMI and AIS^20^.

Sex-stratified analyses identified three susceptibility loci that are significant only in the female meta-analysis. Among the loci, we found an evidence of sex-heterogeneity at the *BOC* locus. *BOC* is a co-receptor for the hedgehog pathway which is induced by 1,25-dihydroxyvitamin D_3_ (1,25(OH)_2_D_3_) in osteocytes^44^. Vitamin D is essential to maintain bone and mineral metabolism. Its status is greatly influenced by gender^45^ and its deficiency is well known to be associated with osteoporosis and autoimmune disease which show significant sexual dimorphism^46^. Thus, *BOC* might be specifically associated with female AIS through the vitamin D metabolism.

In this study, 14 previously unreported AIS susceptibility loci were identified in Japanese, and *TBX1* was identified as one of the AIS susceptibility genes within the loci. Further studies are necessary to validate the association of these susceptibility loci in other ethnic groups. Identifying susceptibility SNPs within the loci and elucidating the function of candidate susceptibility would lead to understanding the etiology and pathogenesis of AIS. Although the mechanism behind sexual dimorphism in AIS remains unknown, the female-specific susceptibility locus we identified will be a key clue to understanding sexual dimorphism in AIS.

## Methods

### Subjects

In GWAS3, as in GWAS1 and GWAS2, the case subjects with a Cobb angle of 10° or greater measured on standing spinal posteroanterior radiographs were recruited from collaborating hospitals^8–10^. All subjects were Japanese and were diagnosed with AIS between the ages of 10 and 18 years by expert scoliosis surgeons^8,47^. Congenital, juvenile, adult-onset scoliosis and scoliosis secondary to some other disorders were excluded. The control subjects were randomly selected from the subjects registered in the BioBank Japan project^48^ (https://biobankjp.org/). For the quality control of GWAS samples, we removed samples with a call rate <0.98. We removed related individuals with PI_HAT > 0.25. PI_HAT is an index of relatedness between two individuals based on identity by descent implemented in PLINK^49^ (https://www.cog-genomics.org/plink2). This filtering of relatedness was applied in each GWAS and combined data of the three GWAS separately and resulted in 102 cases and 806 controls excluded in total. To identify population stratification, we conducted principal component analysis (PCA) for genotype using FastPCA^50^. We excluded outliers from the East Asian cluster (distance from the mean of the cluster should be within 3 SD). There was no overlap of individuals in the three GWASs. Informed consents were obtained from all participants and from the parents of subjects who were minor. The ethical committees at all collaborating institutions and RIKEN approved the study.

### Genotyping and imputation

GWAS3 subjects were genotyped by using the Illumina Human OmniExpress Genotyping BeadChip or a combination of Illumina HumanOmniExpress and HumanExome BeadChips. For quality control of variants, we applied the standard QC measures and excluded those with (i) SNP call rate < 0.99, (ii) MAF < 0.05 and (iii) Hardy–Weinberg equilibrium *P* value ≤ 1.0 × 10^−5^. We also excluded the variants whose allele frequencies had differences of >0.16 between the GWAS dataset and the Asian data in reference panel. For each of the three GWASs, we pre-phased the genotypes using EAGLE and imputed dosages with the 1000 Genomes Project Phase 3 reference panel (May 2013 release; http://www.internationalgenome.org) with 1037 Japanese in-house reference panel using minimac3. For X chromosome, pre-phasing was performed in males and females together, and imputation was performed separately for males and females. Dosages of variants in X chromosomes for males were assigned between 0 and 2. Since variants in the pseudo-autosomal region (PAR) were not contained in the reference panel, we did not analyze PAR. We used variants with an imputation quality score Rsq ≥ 0.3 and MAF ≥ 0.005 for the subsequent association study. The quality control and imputation analysis for three GWASs were processed separately. The number of SNPs through analysis steps is illustrated in Supplementary Table 8.

### GWASs and meta-analysis

Association analyses of autosomes of GWAS1-3 were performed independently in a logistic regression model with top 10 principal components as covariates. The three GWAS were meta-analyzed with inverse-variance method under fixed effect model. For X chromosome, an association analysis was conducted separately for male and female at each GWAS, and meta-analyzed the association results. We filtered variants showing strong heterogeneity (Cochran’s *Q*-test, *P* < 0.0001). This filtering excluded 484 variants in autosomes and 24 variants in X chromosomes. Regional association plots were produced by Locuszoom (http://locuszoom.org). Adjacent genome-wide significant (*P* < 5.0 × 10^−8^) variants were grouped in one locus if they were located within 1 Mb apart from each other. To identify multiple independent signals within the 20 significant loci, we performed a stepwise conditional meta-analysis. We first performed conditional analyses of GWAS1-3 independently, and then combined the results using a fixed-effects model with the inverse-variance method. We repeated this process until the index variants fell below the locus-wide significance threshold of 5.0 × 10^−6^, based on the approximate average number of multiple tests in each locus. Association studies were conducted by plink2 or mach2dat software. We estimated the AIS heritability using the LDSC software. The variance explained by SNPs was calculated based on a liability threshold model by assuming prevalence of AIS as 2.5%. In this model, we assume that subjects have a continuous risk score and that subjects whose score exceed a certain threshold develop AIS.

### Sex-specific GWAS

We separately extracted imputed genotypes of male and female subjects from the GWAS data and applied the same quality control criteria in each GWAS of males and females. We empirically estimated difference in effect sizes in males and females. We randomly generated 100,000 true effect sizes in males and females based on correlation coefficients and standard errors in sex-specific GWAS and compared effect sizes between males and females to compute *P* values. We also calculated statistical power to identify GWAS significant signals in males and females (Methods).

### Power calculation of the current study

We conducted power calculation with use of GeneticsDesign package of R software (https://www.r-project.org/) to detect a signal with *P* value of 5 × 10^−8^ assuming disease prevalence of 3%. Calculation was done for entire dataset, males and females (Supplementary Table 9).

### Estimation of confounding biases using LD score regression

To estimate confounding biases derived from population stratification and cryptic relatedness, we conducted LD score regression^15^. We used LD scores for the East Asian population provided by the LDSC software.

### Functional annotation and eQTL analyses

To characterize associated variants, we used HaploReg v4.1, 3DSNP and RegulomeDB to gain functional annotation of variants in LD (*r*^2^ ≥ 0.8 in EAS of 1KGP3) with the 14 AIS lead variants. LD was calculated using LDlink^51^ (https://ldlink.nci.nih.gov), a web-based application. We searched for the overlaps between these associated variants and promoter and enhancer marks using HaploReg v4.1 setting the source for epigenetic annotation as ChromHMM core 15-state model. We also searched for the overlaps between the associated variants and lead *cis*-eQTL variants in GTEx (release v7). We considered only the *cis*-eQTLs with FDR < 0.05, and listed the variants showing the most significant association for each gene in Supplementary Tables 5 and 6.

### Cell-type specificity analysis

To assess the heritability enrichment in cell types for AIS, we performed stratified LD score regression by combining data from specific annotations of 10 cell-type groups and 220 cell types and four activating histone marks (H3K4me1, H3K4me3, H3K9ac and H3K27ac) from the Roadmap Epigenomics project^17^. The variants with low imputation quality score (Rsq < 0.3) and the variants within the major histocompatibility complex (MHC) region (chromosome 6: 25–34 Mb) were excluded from the regression analysis. We defined significant heritability enrichments as those with *P* < 0.05 after Bonferroni correction.

### Pathway analysis

To investigate biological pathways associated with AIS, we performed PASCAL^19^. PASCAL computes gene scores (max or sum scores) by aggregating SNP *P* values from a GWAS meta-analysis and calculates pathway scores by combining the scores of genes belonging to the same pathways. We used sum statistics and predefined pathway libraries from KEGG, REACTOME and BIOCARTA with default parameters.

### Genetic correlation analysis

To estimate genetic correlations across the 67 traits (61 quantitative traits and 6 diseases), we conducted bivariate LD score regression^15^ using the East Asian LD score and summary statistics of the current GWAS meta-analysis. We excluded SNPs found in the MHC region (chromosome 6: 25–34 Mb) from the analysis because of its complex LD structure. We defined significant genetic correlations as those with FDR < 0.05, calculated via the Benjamini–Hochberg method to correct multiple testing.

### Luciferase assay

We constructed luciferase reporter vectors by cloning *TBX1* promoter (−912~ +63; TBX1-promoter-F: 5′-GTTGGTACCCTCCTCAGTGCTTCCCTTTG-3′ and TBX1-promoter-R: 5′-ACTCTCGAGAGTGTTCCTCCCTCCCTCAC-3′) with oligonucleotides (sense: 5′-TCGATGTCTAATGTACRCACCAGCTCGGA-3′and antisense: 5′-TCGATCCGAGCTGGTGYGTACATTAGACA-3′) containing either risk or non-risk alleles of rs1978060 into the multicloning site of a promoterless pGL4.10[luc2] vector (Promega). We also cloned a cDNA of FOXA2 (FOXA2-F: 5′-GTTAAGCTTGCCACCATGCACTCGGCTTCCAGTAT-3′ and FOXA2-R: 5′-ACTGGATCCAGAGGAGTTCATAATGGGCC-3′) into the multicloning site of p3xFLAG-CMV-14 vector (Sigma-Aldrich) for protein expression in mammalian cell lines. We sequenced the inserts of all constructs. The phRL-SV40 vector (Promega) was used as an internal control to normalize the variation in transfection efficiency. MCF-7 and OUMS-27 cells are generally used for luciferase assay. MCF-7 cells (HTB-22, ATCC) were cultured at 37 °C under 5% CO_2_ in Dulbecco’s modified Eagle’s medium (DMEM) supplemented with 10% fetal bovine serum (FBS), 0.01 mg ml^−1^ human recombinant insulin, 50 U ml^−1^ penicillin and 50 μg ml^−1^ streptomycin. OUMS-27 cells (JCRB Cell Bank) were cultured at 37 °C under 5% CO_2_ in DMEM supplemented with 10% FBS and 50 μg ml^−1^ kanamycin. All cell lines were authenticated by STR analysis and confirmed to be mycoplasma negative. MCF-7 and OUMS-27 cells were seeded in 24-well plates at a density of 5 × 10^4^ and 1 × 10^5^ cells per well, respectively. After 24 h, cells were transfected using TransIT-LT1 (Mirus Bio) according the manufacturer’s instructions. The luciferase activities were measured using the PicaGene Dual Sea Pansy Luminescence kit (Toyo Ink).

### Electrophoretic mobility shift assays

We prepared probes for the risk (G) and non-risk (A) alleles of rs1978060 by annealing 25-bp complementary oligonucleotides (sense: 5′-TGTCTAATGTACRCACCAGCTCGGA-3′ and antisense: 5′-TCCGAGCTGGTGYGTACATTAGACA-3′) and labeling with digoxigenin (DIG)-11-ddUTP (Roche). The nuclear extracts were prepared from MCF-7 and FOXA2-overexpressing OUMS-27 cells. DNA–protein binding reactions were performed using a DIG gel shift kit according to the manufacturer’s instructions (Roche). For competition assays, nuclear extracts were pre-incubated with excess unlabeled probes prior to adding DIG-labeled probes. For a super-shift assay, 2 μg of FOXA2 antibody (Santa Cruz; sc-374376X) was added to the reaction mixture and incubated for 20 min at room temperature. DNA-protein complexes were resolved on 6% DNA retardation gels (Thermo Fisher Scientific), and the signal was detected using a chemiluminescent detection system according to the manufacturer’s instructions (Roche). Uncropped gel images can be found in the Source Data file.

### Reporting summary

Further information on research design is available in the Nature Research Reporting Summary linked to this article.

## Supplementary information

Supplementary informations

Supplementary Data 1

Supplementary Data 2

Supplementary Data 3

Supplementary Data 4

Supplementary Data 5

Supplementary Data 6

Supplementary Data 7

Reporting Summary

Description of Additional Supplementary Files

Peer Review File

Source data

## Data Availability

GWAS summary statistics of AIS are available at JENGER (Japanese ENcyclopedia of GEnetic associations by Riken, http://jenger.riken.jp/). Additional data used in this study are available from the corresponding authors upon reasonable request. The source data underlying Fig. 3a–c and Supplementary Figs. 5a and 5b are provided as a Source Data file.

## References

[1] Kane, W. J. Scoliosis prevalence: a call for a statement of terms. *Clin. Orthop. Relat. Res.* **126**, 43–46 (1977). 598138

[2] Moriyama T (2011). School screening for spine deformity, and epidemiological investigation for spina bifida occulta in Hanshin district (3rd reports).. Shouni No Noushinkei (Nervous System in Children).

[3] Weinstein SL (1999). Natural history. Spine.

[4] Carlson B (2011). ScoliScore AIS prognostic test personalizes treatment for children with spinal curve. Biotechnol. Health..

[5] Cheng JC (2015). Adolescent idiopathic scoliosis. Nat. Rev. Dis. Prim..

[6] Grauers A, Rahman I, Gerdhem P (2012). Heritability of scoliosis. Eur. Spine J..

[7] Miller NH (2005). Identification of candidate regions for familial idiopathic scoliosis. Spine.

[8] Takahashi Y (2011). A genome-wide association study identifies common variants near LBX1 associated with adolescent idiopathic scoliosis. Nat. Genet..

[9] Kou I (2013). Genetic variants in GPR126 are associated with adolescent idiopathic scoliosis. Nat. Genet..

[10] Ogura Y (2015). A functional SNP in BNC2 is associated with adolescent idiopathic scoliosis. Am. J. Hum. Genet..

[11] Sharma S (2015). A PAX1 enhancer locus is associated with susceptibility to idiopathic scoliosis in females. Nat. Commun..

[12] Zhu Z (2015). Genome-wide association study identifies new susceptibility loci for adolescent idiopathic scoliosis in Chinese girls. Nat. Commun..

[13] Zhu Z (2017). Genome-wide association study identifies novel susceptible loci and highlights Wnt/beta-catenin pathway in the development of adolescent idiopathic scoliosis. Hum. Mol. Genet..

[14] Khanshour AM (2018). Genome-wide meta-analysis and replication studies in multiple ethnicities identify novel adolescent idiopathic scoliosis susceptibility loci. Hum. Mol. Genet..

[15] Bulik-Sullivan BK (2015). LD score regression distinguishes confounding from polygenicity in genome-wide association studies. Nat. Genet..

[16] Lowe TG (2000). Etiology of idiopathic scoliosis: current trends in research. J. Bone Jt. Surg. Am..

[17] Roadmap Epigenomics Consortium. (2015). Integrative analysis of 111 reference human epigenomes. Nature.

[18] Finucane HK (2015). Partitioning heritability by functional annotation using genome-wide association summary statistics. Nat. Genet..

[19] Lamparter D, Marbach D, Rueedi R, Kutalik Z, Bergmann S (2016). Fast and rigorous computation of gene and pathway scores from SNP-based summary statistics. PLoS Comput. Biol..

[20] Akiyama M (2017). Genome-wide association study identifies 112 new loci for body mass index in the Japanese population. Nat. Genet..

[21] Kanai M (2018). Genetic analysis of quantitative traits in the Japanese population links cell types to complex human diseases. Nat. Genet..

[22] Bulik-Sullivan B (2015). An atlas of genetic correlations across human diseases and traits. Nat. Genet..

[23] Raggio CL (2006). Sexual dimorphism in adolescent idiopathic scoliosis. Orthop. Clin. North Am..

[24] GTEx Consortium. (2015). Human genomics. The Genotype-Tissue Expression (GTEx) pilot analysis: multitissue gene regulation in humans. Science.

[25] Yagi H (2003). Role of TBX1 in human del22q11.2 syndrome. Lancet.

[26] Morava E, Lacassie Y, King A, Illes T, Marble M (2002). Scoliosis in velo-cardio-facial syndrome. J. Pediatr. Orthop..

[27] Bassett AS (2005). Clinical features of 78 adults with 22q11 deletion syndrome. Am. J. Med Genet. A.

[28] Homans JF (2019). Scoliosis in association with the 22q11.2 deletion syndrome: an observational study. Arch. Dis. Child.

[29] Jerome LA, Papaioannou VE (2001). DiGeorge syndrome phenotype in mice mutant for the T-box gene, Tbx1. Nat. Genet..

[30] Lindsay EA (2001). Tbx1 haploinsufficieny in the DiGeorge syndrome region causes aortic arch defects in mice. Nature.

[31] Wu N (2015). TBX6 null variants and a common hypomorphic allele in congenital scoliosis. N. Engl. J. Med..

[32] Kou I (2018). A multi-ethnic meta-analysis confirms the association of rs6570507 with adolescent idiopathic scoliosis. Sci. Rep..

[33] Ogura Y (2018). An international meta-analysis confirms the association of BNC2 with adolescent idiopathic scoliosis. Sci. Rep..

[34] Haller G (2018). A missense variant in SLC39A8 is associated with severe idiopathic scoliosis. Nat. Commun..

[35] Siu King Cheung C (2003). Abnormal peri-pubertal anthropometric measurements and growth pattern in adolescent idiopathic scoliosis: a study of 598 patients. Spine.

[36] Ramirez M (2013). Body composition in adolescent idiopathic scoliosis. Eur. Spine J..

[37] MacEwen GD, Winter RB, Hardy JH (1972). Evaluation of kidney anomalies in congenital scoliosis. J. Bone Jt. Surg. Am..

[38] Drvaric DM (1987). Congenital scoliosis and urinary tract abnormalities: are intravenous pyelograms necessary?. J. Pediatr. Orthop..

[39] Yamagishi H (2003). Tbx1 is regulated by tissue-specific forkhead proteins through a common Sonic hedgehog-responsive enhancer. Genes Dev..

[40] Hu T (2004). Tbx1 regulates fibroblast growth factors in the anterior heart field through a reinforcing autoregulatory loop involving forkhead transcription factors. Development.

[41] Frayling TM (2007). A common variant in the FTO gene is associated with body mass index and predisposes to childhood and adult obesity. Science.

[42] Dina C (2007). Variation in FTO contributes to childhood obesity and severe adult obesity. Nat. Genet..

[43] Okada Y (2012). Common variants at CDKAL1 and KLF9 are associated with body mass index in east Asian populations. Nat. Genet..

[44] St John HC (2014). The osteoblast to osteocyte transition: epigenetic changes and response to the vitamin D3 hormone. Mol. Endocrinol..

[45] Verdoia M (2015). Impact of gender difference on vitamin D status and its relationship with the extent of coronary artery disease. Nutr. Metab. Cardiovasc. Dis..

[46] Yoshimura N (2013). Profiles of vitamin D insufficiency and deficiency in Japanese men and women: association with biological, environmental, and nutritional factors and coexisting disorders: the ROAD study. Osteoporos. Int..

[47] Ogura Y (2013). A replication study for association of 5 single nucleotide polymorphisms with curve progression of adolescent idiopathic scoliosis in Japanese patients. Spine.

[48] Nakamura Y (2007). The BioBank Japan Project. Clin. Adv. Hematol. Oncol..

[49] Purcell S (2007). PLINK: a tool set for whole-genome association and population-based linkage analyses. Am. J. Hum. Genet.

[50] Galinsky KJ (2016). Fast principal-component analysis reveals convergent evolution of ADH1B in Europe and East Asia. Am. J. Hum. Genet..

[51] Machiela MJ, Chanock SJ (2015). LDlink: a web-based application for exploring population-specific haplotype structure and linking correlated alleles of possible functional variants. Bioinformatics.

