# Supplementary material for: Genome-wide association study identifies 14 previously unreported susceptibility loci for adolescent idiopathic scoliosis in Japanese
Source: Nat Commun. 2019 Aug 15;10:3685. doi: 10.1038/s41467-019-11596-w (PMC6695451; doi:10.1038/s41467-019-11596-w)
Supplement: Supplementary file 1 — Supplementary informations [file 41467_2019_11596_MOESM1_ESM.pdf]

Supplementary Information

Genome-wide association study identifies 14 previously unreported susceptibility loci for adolescent idiopathic scoliosis in Japanese

Kou, I. *et al.*

Table of contents

Supplementary Figures

Supplementary Figure 1. Overview of the genome-wide association studies (GWASs) for adolescent idiopathic scoliosis in Japanese 2

Supplementary Figure 2. Regional association plots of the 14 previously unreported AIS loci 3

Supplementary Figure 3. Regional association plots of the conditional analysis 7

Supplementary Figure 4. Enrichment of cell-type groups 8

Supplementary Figure 5. Allelic difference of functional variant, rs1978060 in chromosome 22q11.21 9

Supplementary Tables

Supplementary Table 1. Comparison of minor allele frequencies between Japanese and European 10

Supplementary Table 2. Independent signals identified by conditional analysis 11

Supplementary Table 3. Six secondary signals found by GCTA-COJO 12

Supplementary Table 4. Heritability enrichment of the 10 cell-type groups 13

Supplementary Table 5. Summary of eQTL variants correlated with previously unreported AIS signals ($r^2 > 0.8$) .14

Supplementary Table 6. Summary of eQTL variants correlated with female AIS signals ($r^2 > 0.8$) 15

Supplementary Table 7. Description of the genes identified in this study 16

Supplementary Table 8. Numbers of variants in three batches in the analyses 20

Supplementary Table 9. Statistical power to obtain GWAS significance in the current study 21

Supplementary Note 1 22

Statistical power of the current study....22

Conditional analyses with the use of GCTA-COJO 22

Supplementary Reference 24

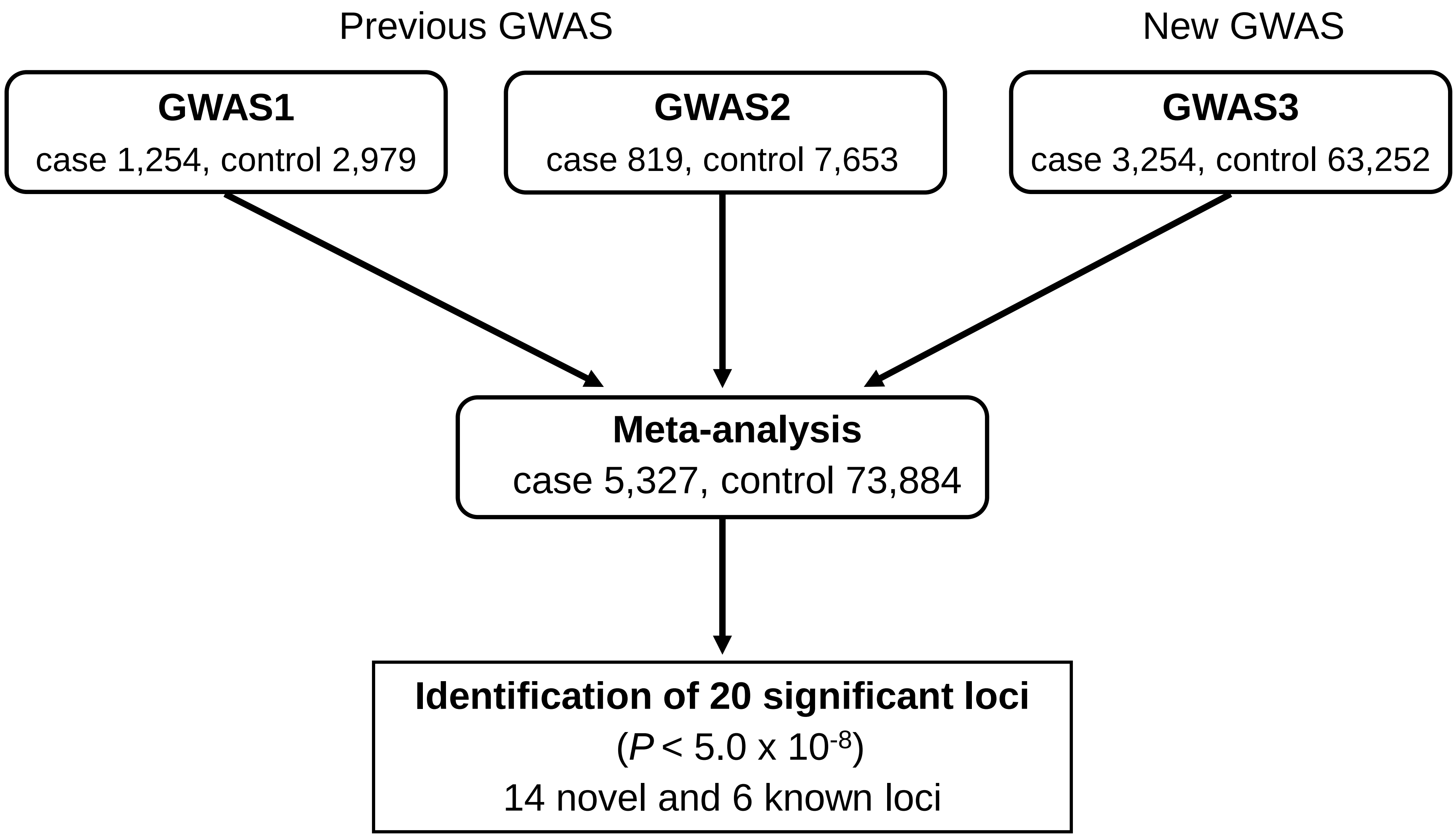

Supplementary Figure 1. Overview of the genome-wide association studies (GWASs) for adolescent idiopathic scoliosis in Japanese.

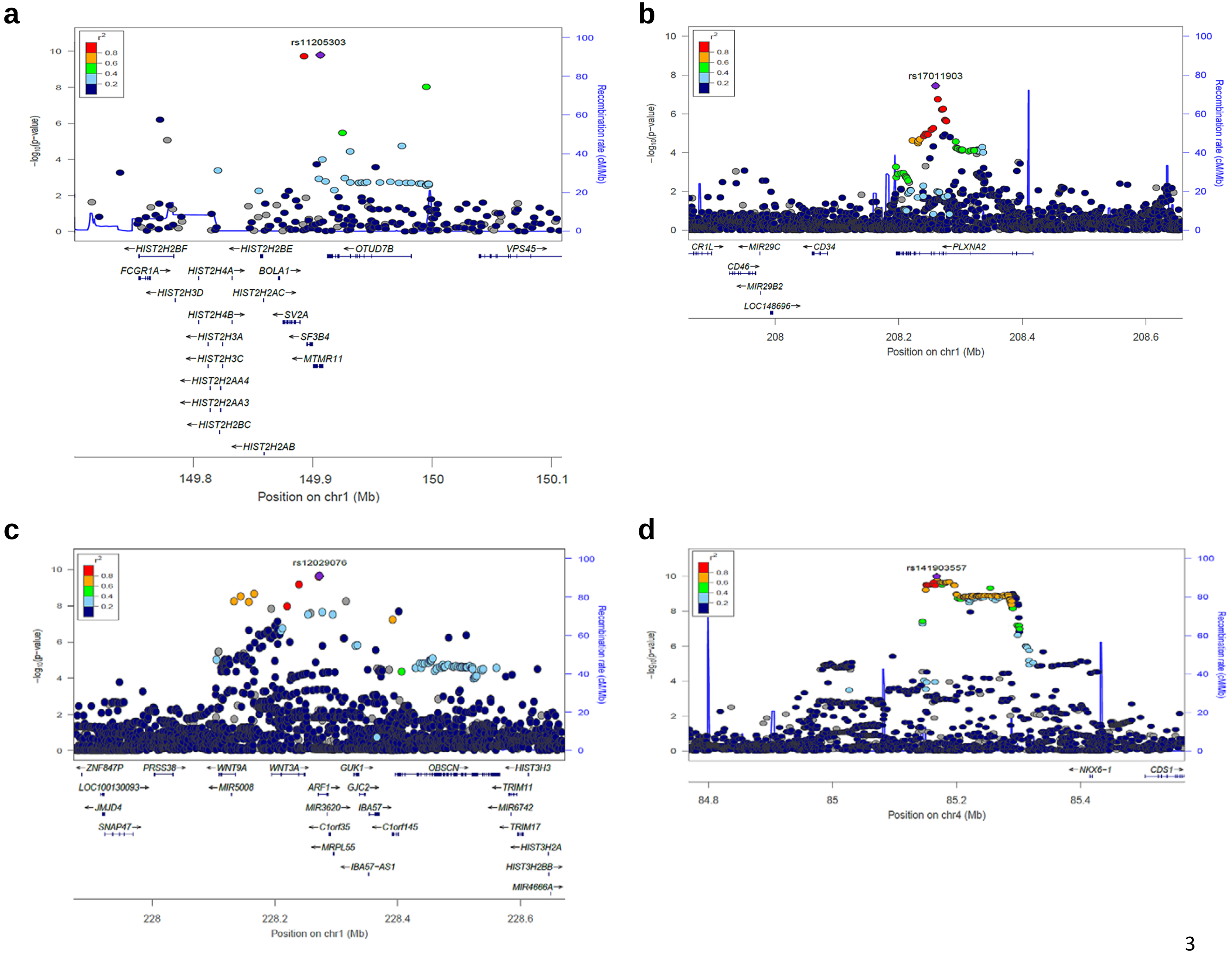
a
rs11205303
r²
0.8
0.6
0.4
0.2
-log10(p-value)
Recombination rate (cM/Mb)
HIST2H2BF
HIST2H2BE
OTUD7B
VPS45
FCGR1A
HIST2H4A
BOLA1
HIST2H3D
HIST2H2AC
HIST2H4B
SV2A
HIST2H3A
SF3B4
HIST2H3C
MTMR11
HIST2H2AA4
HIST2H2AA3
HIST2H2BC
HIST2H2AB
149.8
149.9
150
150.1
Position on chr1 (Mb)
b
rs17011903
CR1L
MIR29C
CD34
PLXNA2
CD46
MIR29B2
LOC148696
208
208.2
208.4
208.6
Position on chr1 (Mb)
c
rs12029076
ZNF847P
PRSS38
WNT9A
WNT3A
GUK1
OBSCN
HIST3H3
LOC100130093
MIR5008
ARF1
GJC2
TRIM11
JMJD4
MIR3620
IBA57
MIR6742
SNAP47
C1orf35
C1orf145
TRIM17
MRPL55
HIST3H2A
IBA57-AS1
HIST3H2BB
MIR4666A
228
228.2
228.4
228.6
Position on chr1 (Mb)
d
rs141903557
NKX6-1
CDS1
84.8
85
85.2
85.4
Position on chr4 (Mb)

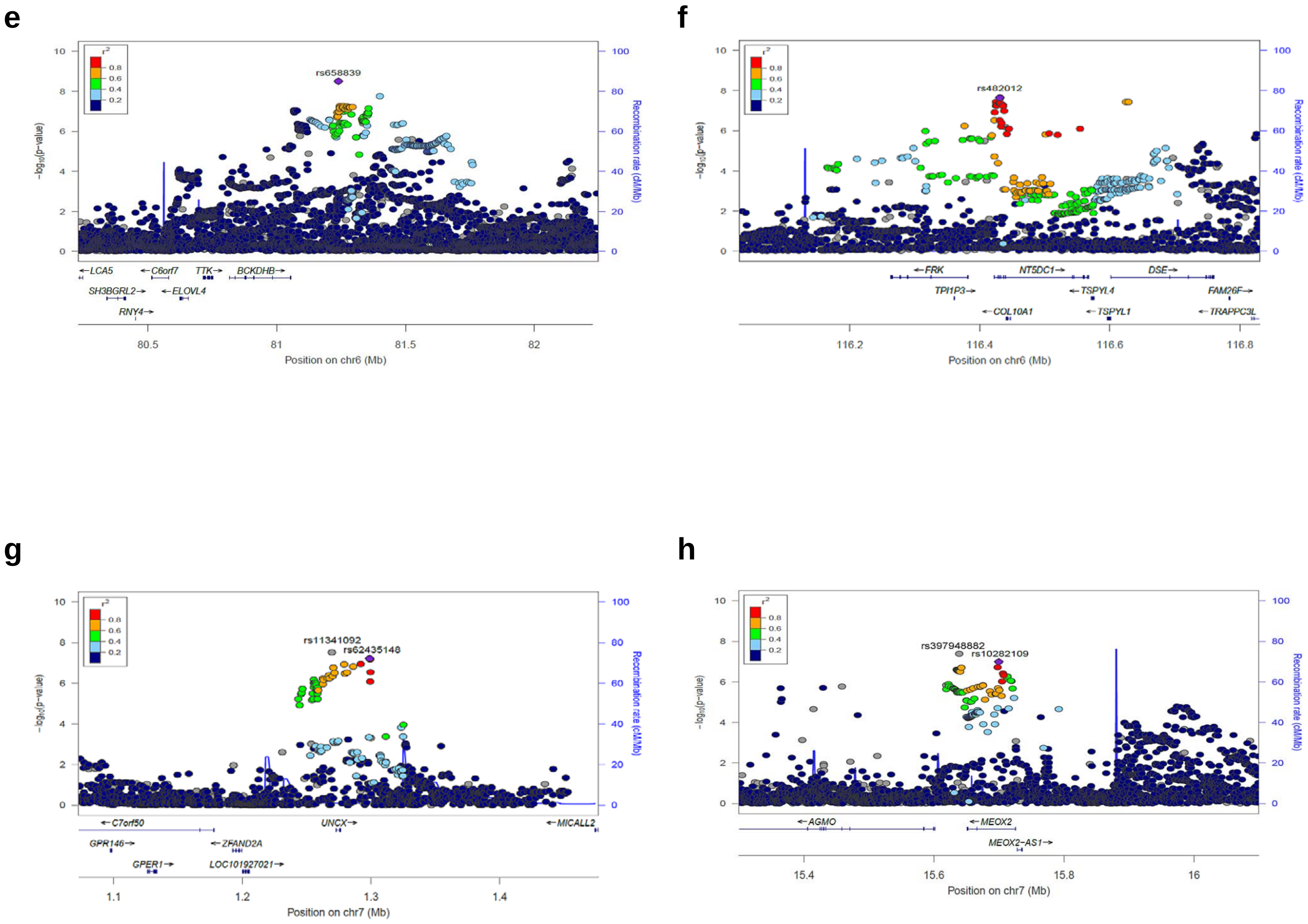
e
rs658839
LCA5
C6orf7
TTK
BCKDHB
SH3BGRL2
ELOVL4
RNY4
Position on chr6 (Mb)
Recombination rate (cM/Mb)
f
rs482012
FRK
NT5DC1
DSE
TPI1P3
TSPYL4
FAM26F
COL10A1
TSPYL1
TRAPPC3L
Position on chr6 (Mb)
Recombination rate (cM/Mb)
g
rs11341092
rs62435148
C7orf50
UNCX
MICALL2
GPR146
ZFAND2A
GPER1
LOC101927021
Position on chr7 (Mb)
Recombination rate (cM/Mb)
h
rs397948882
rs10282109
AGMO
MEOX2
MEOX2-AS1
Position on chr7 (Mb)
Recombination rate (cM/Mb)

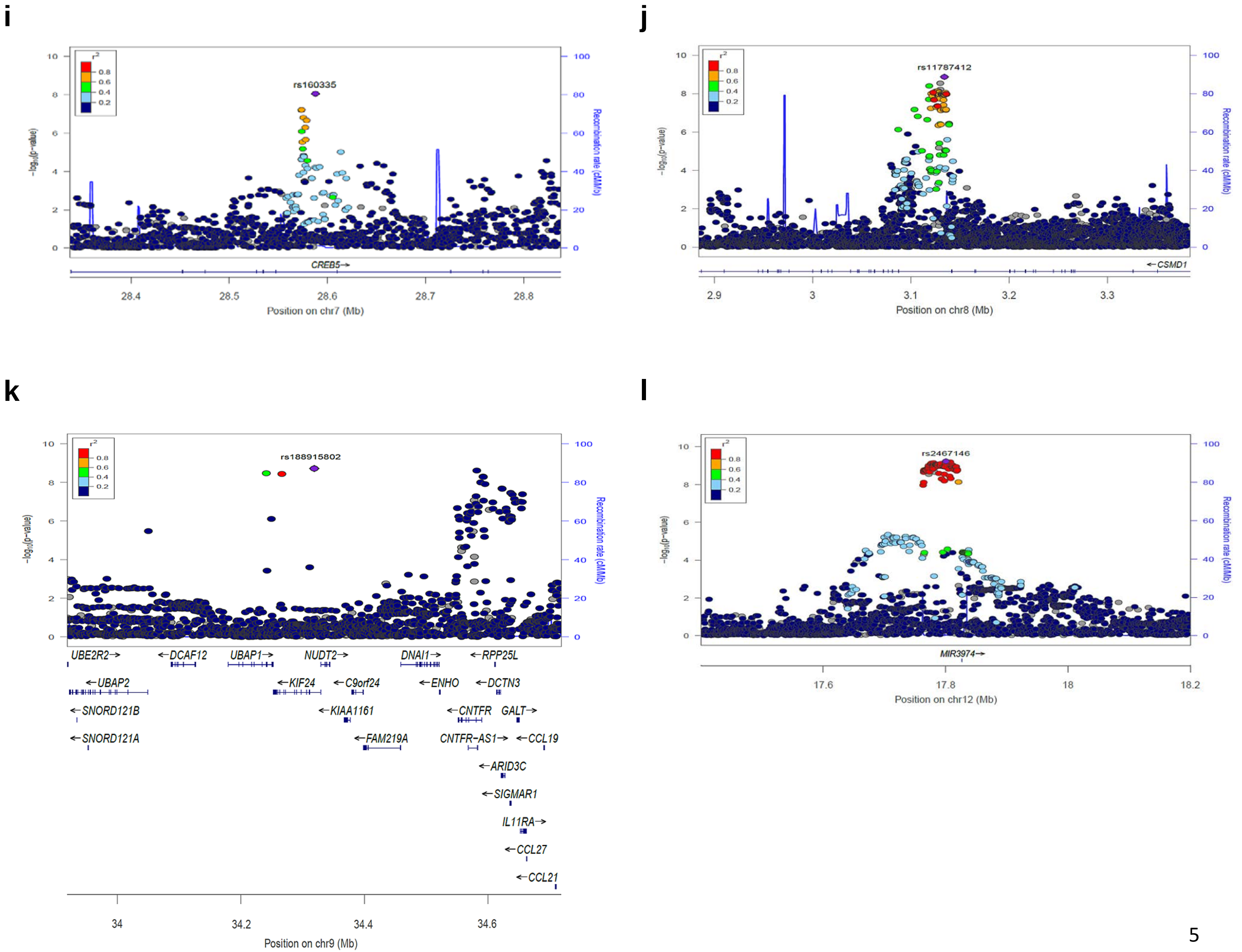
i
rs160335
r²
0.8
0.6
0.4
0.2
-log10(p-value)
Recombination rate (cM/Mb)
CREB5→
28.4
28.5
28.6
28.7
28.8
Position on chr7 (Mb)
j
rs11787412
←CSMD1
2.9
3
3.1
3.2
3.3
Position on chr8 (Mb)
k
rs188915802
UBE2R2→
←DCAF12
UBAP1→
NUDT2→
DNAI1→
←RPP25L
←UBAP2
←KIF24
←C9orf24
←ENHO
←DCTN3
←SNORD121B
←KIAA1161
←CNTFR
GALT→
←SNORD121A
←FAM219A
CNTFR−AS1→
←CCL19
←ARID3C
←SIGMAR1
IL11RA→
←CCL27
←CCL21
34
34.2
34.4
34.6
Position on chr9 (Mb)
l
rs2467146
MIR3974→
17.6
17.8
18
18.2
Position on chr12 (Mb)

m

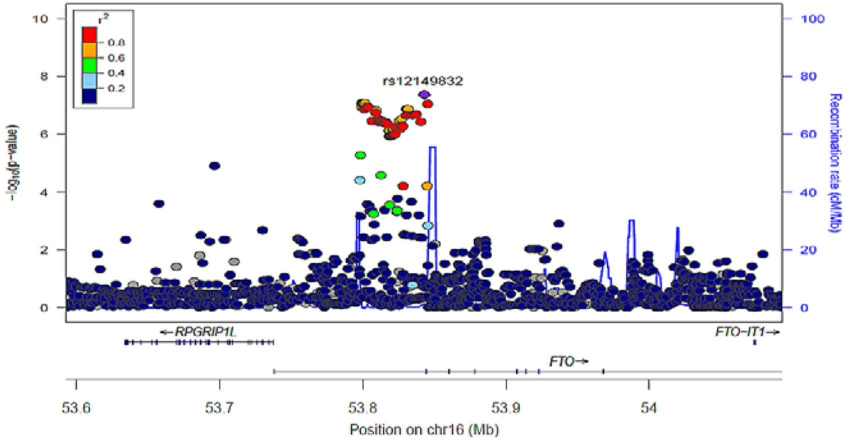

n

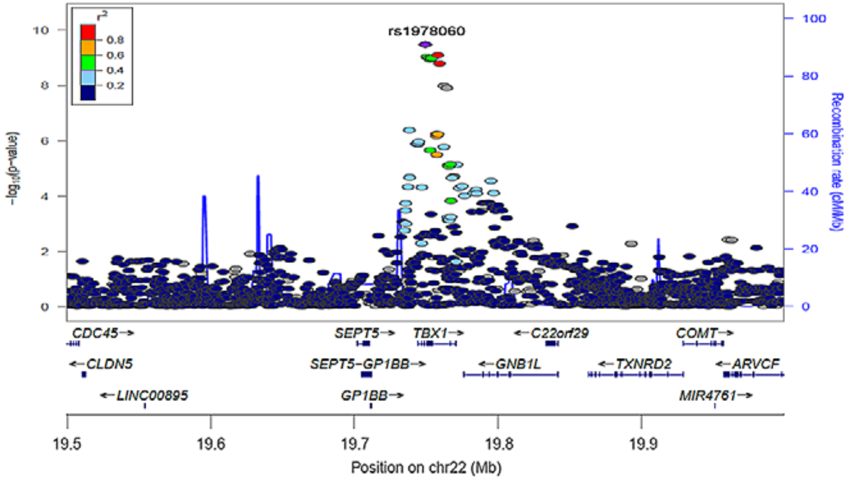

Supplementary Figure 2. Regional association plots of the 14 previously unreported AIS loci. Each plot shows the most associated SNP (lead SNP: purple diamond) and its genomic position. (**a**) 1q21.2. (**b**) 1q32.2. (**c**) 1q42.13. (**d**) 4q21.23. (**e**) 6q14.1. (**f**) 6q22.1. (**g**) 7p22.3. (**h**) 7p21.2. (**i**) 7p15.1. (**j**) 8p23.2. (**k**) 9p13.3. (**l**) 12p12.3. (**m**) 16q12.2. (**n**) 22q11.21. x axis, genomic position; y axis, $-\log_{10} P$. The lead SNPs (gray dots), rs11341092 at the 7p22.3 (g) and rs397948882 at the 7p21.2 (h) were deletion/insertion polymorphisms. Because their linkage disequilibrium data were not available in LocusZoom, the next most significantly associated SNPs (rs62435148 and rs10282109) were used for each regional association plot.

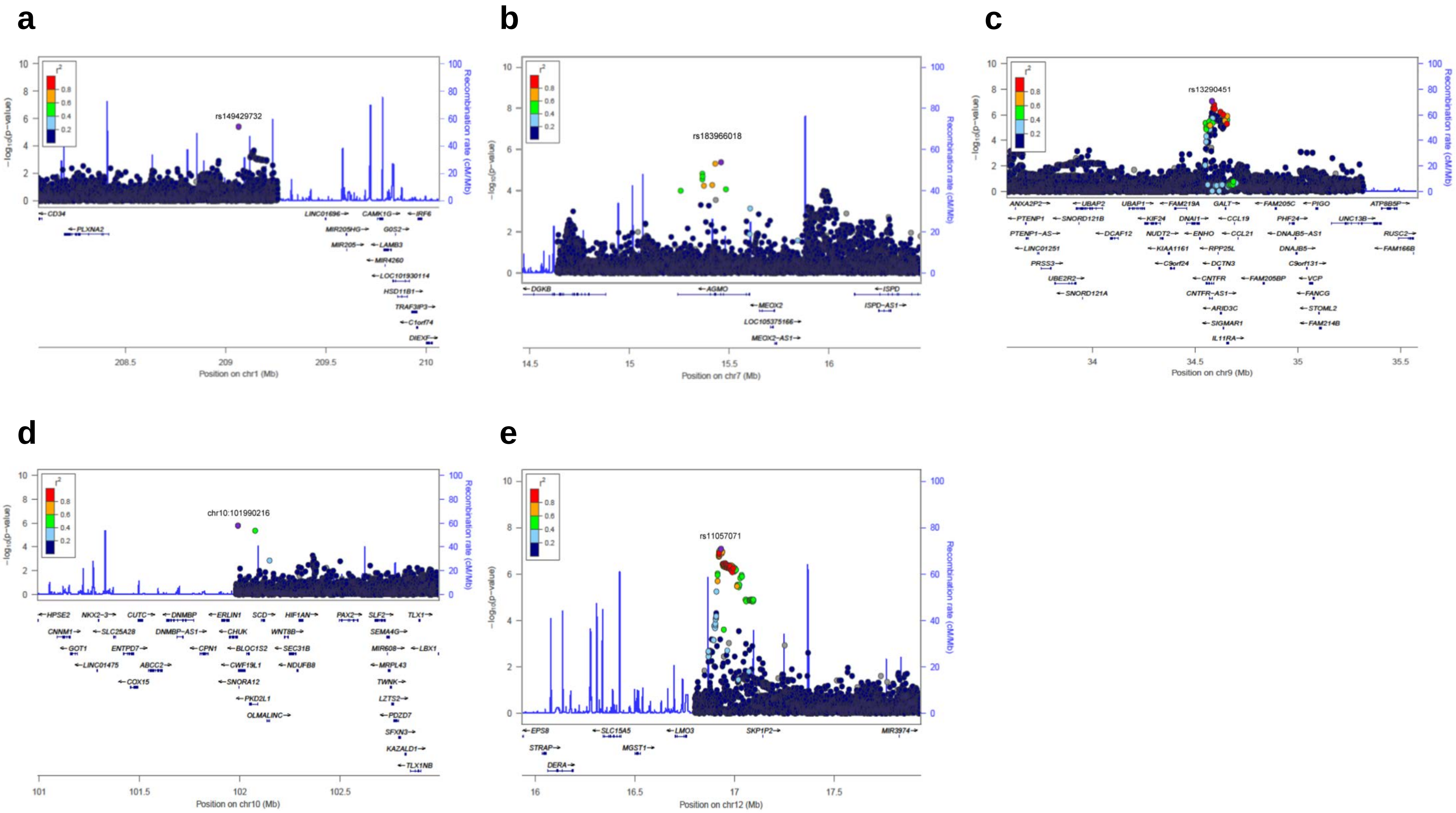

Supplementary Figure 3. Regional association plots of the conditional analysis. Each plot shows the result after conditioning on the lead SNP of each locus. (**a**) 1q32.2. (**b**) 7p21.2. (**c**) 9p13.3. (**d**) 10q24.31. (**e**) 12p12.3. x axis, genomic position; y axis, $-\log_{10}$ *P.* The secondary signal at each locus is represented as a purple dot.

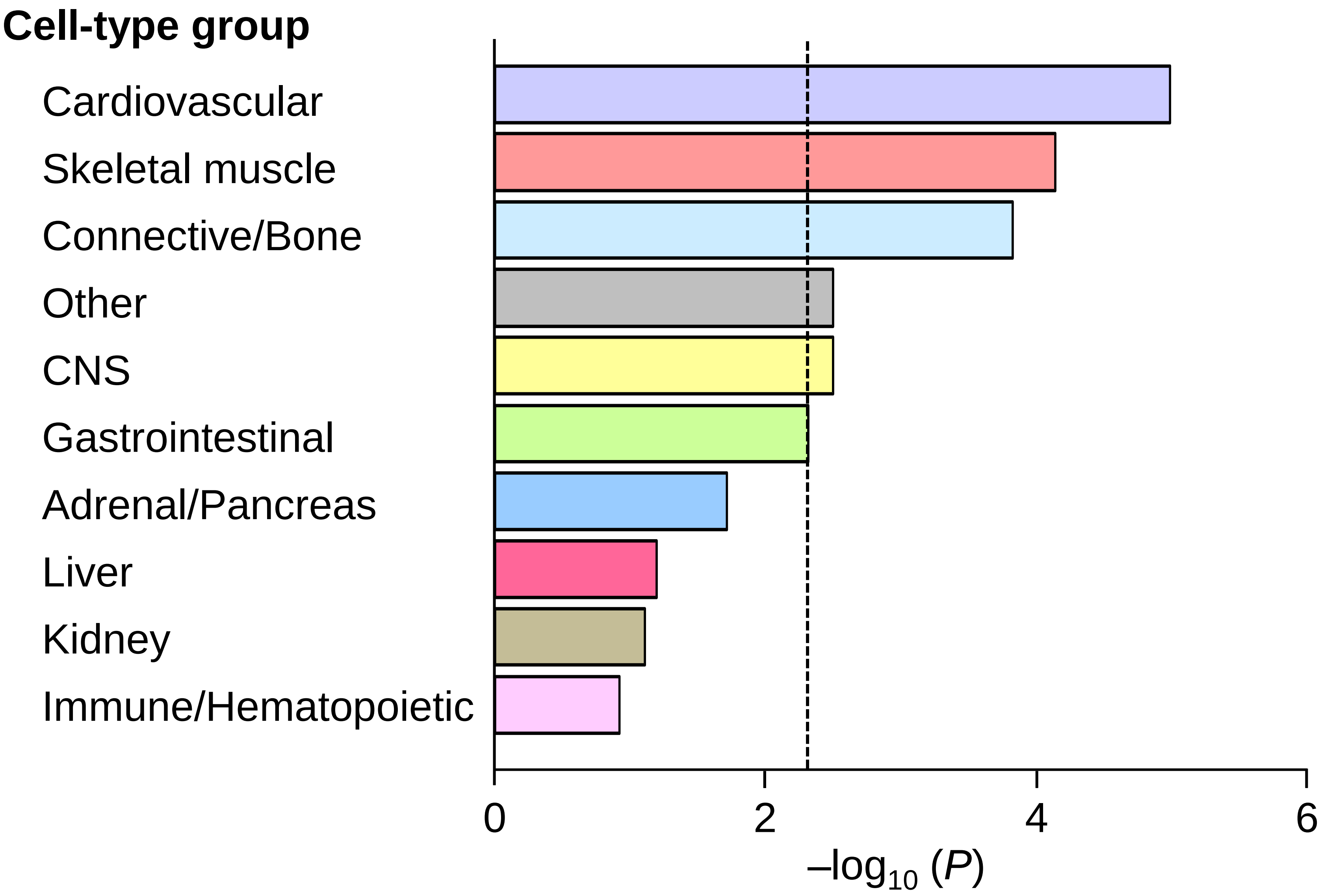

Supplementary Figure 4. Enrichment of cell-type groups. The significant enrichment was observed in six cell type groups. The black dashed lines at $-\log_{10}(P)=2.3$ is the cut off for the Bonferroni significance.

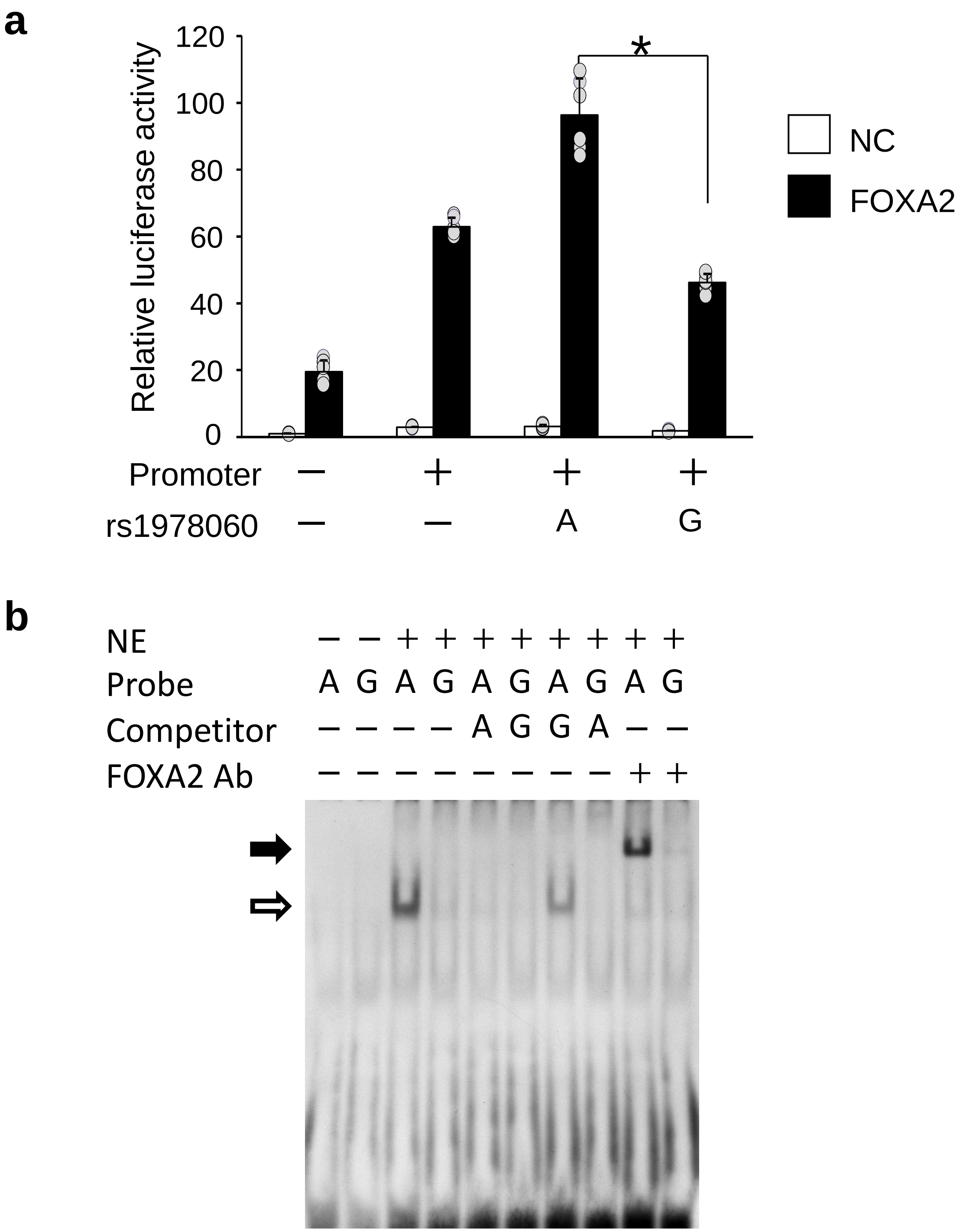

Supplementary Figure 5. Allelic difference of functional variant, rs1978060 in chromosome. 22q11.21. (**a**) Reporter assays in OUMS-27 cells. There was a significantly decreased transcriptional activity for the risk G-allele of rs1978060 compared to the non-risk A-allele. Error bars show standard deviation (S.D.) for each variant. Asterisks indicate statistically significant changes in paired comparison (t-test $P < 0.01$). $n = 2$ independent experiments. (**b**) Electrophoretic mobility shift assays with nuclear extracts from *FOXA2* overexpressing OUMS-27 cells. There was specific band for the A-allele probe (lane 3, white arrow) of rs1978060. Competition analyses were performed using an excess of the unlabeled A-allele (lane 5 and 8) and G-allele (lane 6 and 7) probes as competitors. Lane 9 and 10: A super-shift assay using the FOXA2 antibody. The black arrow indicates a super-shifted FOXA2 complex in lane 9. Source data are provided as a Source Data file.

Supplementary Table 1 Comparison of minor allele frequencies between Japanese and European

SNP	Chr.	Pos.	MA_{JPN}	OR	95%CI	MAF	
						JPN[a]	EUR[b]
**Previously unreported loci**							
rs11205303	1q21.2	149906413	C	1.17	1.11-1.23	0.209	0.369
rs17011903	1q32.2	208259531	A	1.20	1.13-1.28	0.096	0.004
rs12029076	1q42.13	228272687	C	0.85	0.80-0.89	0.222	0.201
rs141903557	4q21.23	85168056	C	1.33	1.22-1.45	0.047	0.009
rs658839	6q14.1	81228722	A	0.88	0.84-1.92	0.492	0.530
rs482012	6q22.1	116430533	G	0.88	0.84-0.92	0.284	0.400
rs11341092	7p22.3	1269592	AC	1.14	1.09-1.19	0.314	0.178
rs397948882[c]	7p21.2	15636869	A	1.20	1.12-1.28	0.099	0.374
rs160335	7p15.1	28587817	A	0.89	0.85-0.92	0.495	0.175
rs11787412	8p23.2	3134239	A	1.14	1.09-1.18	0.385	0.346
rs188915802	9p13.3	34318683	T	1.66	1.41-1.96	0.013	0.000
rs2467146	12p12.3	17800607	G	0.87	0.83-0.91	0.327	0.227
rs12149832	16q12.2	53842908	A	0.86	0.82-0.91	0.210	0.171
rs1978060	22q11.21	19749525	G	1.16	1.11-1.22	0.465	0.632
**Previously reported loci**							
rs9389985	6q24.1	142653898	G	1.21	1.16-1.26	0.427	0.320
rs7028900	9p22.2	16690612	C	1.20	1.15-1.25	0.419	0.771
rs144131194	9q34.2	136145993	A	0.87	0.83-0.90	0.452	0.370
rs11190870	10q24.31	102979207	C	0.66	0.63-0.69	0.440	0.409
rs2194285	16q23.3	82894817	G	1.19	1.12-1.27	0.115	0.380
rs6047716	20p11.22	21894005	C	1.15	1.11-1.20	0.475	0.444
**Female AIS loci**							
rs142502288	1q23.3	162450931	G	1.52	1.31-1.76	0.022	0.000
rs545608	1q25.2	177899121	C	0.86	0.82-0.91	0.267	0.186
rs73235136	3q13.2	112951529	C	1.15	1.10-1.20	0.465	0.813

Chr., chromosome; Pos., genomic position (GRCh37/hg19); MA_{JPN}, minor allele in Japanese samples of the present study; OR, Odds ratio; CI, confidence interval; MAF, minor allele frequency; JPN, Japanese; EUR, European.

[a]MAF in Japanese samples of the present study; [b]MAF in European samples of 1000 Genomes Project; [c]rs397948882 in dbSNP build142 was shown as a rs57982636 in dbSNP build 151.

Supplementary Table 2 Independent signals identified by conditional analysis

Position (hg19)	SNP	Condition SNP	RA	Unconditional		Conditional		Variance[a]
				P value	OR (95% CI)	*P* value	OR (95% CI)	
1:209065127	rs149429732	rs17011903	T	3.12E-06	2.23 (1.59-3.12)	3.80E-06	2.21 (1.58-3.10)	0.0015
7:15458539	rs183966018	rs397948882	T	1.63E-06	1.74 (1.39-2.18)	4.05E-06	1.70 (1.36-2.13)	0.0008
9:34581819	rs13290451	rs188915802	T	2.49E-09	1.13 (1.09-1.18)	8.54E-08	1.12 (1.07-1.17)	0.0012
10:101990216	chr10:101990216	rs11190870	T	5.54E-07	1.91 (1.48-2.47)	1.57E-06	1.87 (1.45-2.41)	0.0011
12:16933039	rs11057071	rs2467146	A	3.96E-08	1.12 (1.05-1.20)	8.21E-08	1.12 (1.07-1.17)	0.0011

RA, risk allele; OR, odds ratio; CI, confidence interval.

[a]The proportion of variance explained by the significant SNP.

Supplementary Table 3 Six secondary signals found by GCTA-COJO

Position (hg19)	SNP	RA	RA frequency	OR (95% CI)	*P* value
1:209065127	rs149429732	T	0.0058	2.25 (1.61-3.15)	2.42E-06
7:15429577	rs143289197	C	0.0079	1.71 (1.38-2.12)	1.18E-06
9:15952305	rs78223574	A	0.89	1.18 (1.10-1.27)	3.61E-06
9:34581819	rs13290451	T	0.54	1.12 (1.08-1.18)	1.56E-08
10:101990216	chr10:101990216	T	0.0070	1.89 (1.47-2.44)	8.60E-07
12:16933039	rs11057071	A	0.56	1.12 (1.08-1.16)	5.77E-08

RA, risk allele; OR, odds ratio; CI, confidence interval.

Supplementary Table 4 Heritability enrichment of the 10 cell-type groups

Group	Prop. SNP	Prop. h^2	Prop. h^2 SE	Enrichment	Enrichment SE	Enrichment *P*
Cardiovascular	1.10E-01	3.71E-01	5.87E-02	3.38	0.53	1.07E-05
Skeletal muscle	1.02E-01	3.13E-01	5.19E-02	3.06	0.51	7.53E-05
Connective/Bone	1.13E-01	3.18E-01	5.45E-02	2.82	0.48	1.55E-04
Other	2.00E-01	4.07E-01	6.98E-02	2.03	0.35	3.25E-03
CNS	1.48E-01	2.97E-01	5.05E-02	2.01	0.34	3.25E-03
Gastrointestinal	1.68E-01	3.52E-01	6.45E-02	2.10	0.38	4.96E-03
Adrenal/Pancreas	9.21E-02	2.13E-01	5.12E-02	2.31	0.56	1.96E-02
Liver	7.16E-02	1.45E-01	3.94E-02	2.03	0.55	6.45E-02
Kidney	4.23E-02	1.22E-01	4.51E-02	2.89	1.07	7.90E-02
Immune/Hematopoietic	2.33E-01	3.37E-01	6.72E-02	1.45	0.29	1.21E-01

Prop., proportion; SE, standard errors.

Supplementary Table 5 Summary of eQTL variants correlated with previously unreported AIS signals (r^2 > 0.8)

SNP[a]	Variant ID	Ref	Alt	Lead SNP	r^2	Tissue	Gene	GENCODE ID	NES[b]	P_{eQTL}
rs11205277	1:149892872_A_G	A	G	rs11205303	0.99	Cells - Transformed fibroblasts	RP11-196G18.22	ENSG00000261716.1	0.24	1.5E-08
rs36180369	1:228315703_CCTAT_C	CCTAT	C	rs12029076	0.82	Cells - Transformed fibroblasts	RP5-1139B12.3	ENSG00000269934.1	0.41	3.0E-11
rs73092904	1:228239301_C_T	C	T	rs12029076	0.95	Cells - Transformed fibroblasts	RP5-1139B12.2	ENSG00000269890.1	0.45	1.0E-08
rs36180369	1:228315703_CCTAT_C	CCTAT	C	rs12029076	0.82	Esophagus - Mucosa	OBSCN	ENSG00000154358.15	-0.32	1.0E-08
rs10916258	1:228219882_C_A	C	A	rs12029076	0.89	Thyroid	WNT9A	ENSG00000143816.7	-0.25	2.8E-06
rs12029076	1:228272687_G_C	G	C	rs12029076	1.00	Thyroid	C1orf145	ENSG00000162913.9	0.22	5.6E-05
rs9488836	6:116435795_C_T	C	T	rs482012	0.96	Muscle - Skeletal	FRK	ENSG00000111816.6	-0.30	3.5E-17
rs1064583	6:116446576_A_G	A	G	rs482012	0.92	Muscle - Skeletal	NT5DC1	ENSG00000178425.9	-0.23	1.5E-13
rs3051954	6:116439744_C_CAT	C	CAT	rs482012	0.92	Cells - Transformed fibroblasts	TSPYL4	ENSG00000187189.9	0.28	3.1E-16
rs3051954	6:116439744_C_CAT	C	CAT	rs482012	0.92	Testis	DSE	ENSG00000111817.12	0.37	2.8E-10
rs509236	6:116432703_T_C	T	C	rs482012	0.92	Testis	FAM26E	ENSG00000178033.5	-0.27	2.3E-05
rs2228547	6:116441646_C_G	C	G	rs482012	0.88	Testis	TPI1P3	ENSG00000186743.2	0.38	5.6E-06
rs9488837	6:116435957_G_A	G	A	rs482012	0.96	Testis	FAM26F	ENSG00000188820.8	0.36	1.8E-05
rs4724805	7:1279795_C_T	C	T	rs11341092	0.80	Testis	AC073094.4	ENSG00000233082.1	-0.34	7.1E-06
rs2467168	12:17770833_T_C	T	C	rs2467146	0.97	Testis	RP11-606D9.1	ENSG00000256389.1	-0.35	1.5E-13
rs56094641	16:53806453_A_G	A	G	rs12149832	0.90	Muscle - Skeletal	FTO	ENSG00000140718.14	0.14	1.6E-05
rs737869	22:19759437_G_C	G	C	rs1978060	0.81	Nerve - Tibial	C22orf29	ENSG00000215012.4	-0.32	3.3E-11
rs737869	22:19759437_G_C	G	C	rs1978060	0.81	Nerve - Tibial	AC000089.3	ENSG00000235776.2	-0.25	8.6E-06
rs737869	22:19759437_G_C	G	C	rs1978060	0.81	Nerve - Tibial	AC000078.5	ENSG00000232926.1	0.22	4.8E-05
rs2238777	22:19758228_A_G	A	G	rs1978060	0.82	Thyroid	GNB1L	ENSG00000185838.9	0.22	2.6E-05
rs1978060	22:19749525_A_G	A	G	rs1978060	1.00	Prostate	TBX1	ENSG00000184058.8	-0.76	5.2E-12

Ref, reference allele; Alt, alternate allele; NES, normalized effect size.

[a]Among the variants in high LD (r^2 > 0.8 in EAS) with novel AIS signals, the strongest cis-eQTL variant for each candidate gene was shown as a representative marker.

[b]NES is defined as the slope of the linear regression, and is computed as the effect of the Alt relative to the Ref in the human genome reference GRCh37/hg19.

Supplementary Table 6 Summary of eQTL variants correlated with female AIS signals (r^2 > 0.8)

SNP[a]	Variant ID	Ref	Alt	Lead SNP	r^2	Tissue	Gene	GENCODE ID	NES[b]	P_{eQTL}
rs4682481	3_112983855_C_T_b37	C	T	rs73235136	0.85	Nerve - Tibial	BOC	ENSG00000144857.10	0.31	1.1E-12
rs73235142	3_112966511_G_C_b37	G	C	rs73235136	0.84	Testis	WDR52	ENSG00000206530.4	-0.30	1.3E-08
rs4682478	3_112966721_T_C_b37	T	C	rs73235136	0.84	Testis	WDR52-AS1	ENSG00000243849.1	-0.22	4.4E-06

Ref, reference allele; Alt, alternate allele; NES, normalized effect size.

[a]Among the variants in high LD (r^2 > 0.8 in EAS) with novel AIS signals, the strongest cis-eQTL variant for each candidate gene was shown as a representative marker.

[b]NES is defined as the slope of the linear regression, and is computed as the effect of the Alt relative to the Ref in the human genome reference GRCh37/hg19.

Supplementary Table 7 Description of the genes identified in this study

Gene name	Long gene name	Gene function
**The nearest genes in the previously unreported AIS loci**		
MTMR11	myotubularin related protein 11	*MTMR11* is a Protein Coding gene. The protein belongs to the non-receptor class myotubularin subfamily but has been suggested to have no phosphatase activity because it lacks a conserved active site cysteine residue (UniProt).
PLXNA2	plexin A2	*PLXNA2* encodes a member of the plexin-A family of semaphorin co-receptors. A subset of semaphorins are recognized by plexin-A/neuropilin transmembrane receptor complexes, triggering a cellular signal transduction cascade that leads to axon repulsion. PLXNA2 recognizes secreted or membrane-bound semaphorin 3A, which is implicated in neural regulation of bone metabolism (RefSeq, PMID: 16932874).
ARF1	ADP ribosylation factor 1	*ARF1* is a member of the human ARF gene family, which encodes a small guanine nucleotide binding protein. ARF1 is localized to the Golgi apparatus and has a central role in intra-Golgi transport. GTP-bound ARF1 interacts with PICK1 and its function is linked to AMPA receptor trafficking, regulation of synaptic plasicity of excitatory synapses and spine shrinkage during long-term depression (RefSeq, PMID: 23889934).
LOC101928978	Uncharacterized LOC101928978	*LOC101928978 (Uncharacterized LOC101928978)* is an RNA Gene, and is affiliated with the ncRNA class.
BCKDHB	branched chain keto acid dehydrogenase E1 subunit beta	*BCKDHB* encodes the E1 beta subunit of branched-chain keto acid dehydrogenase, which is a multienzyme complex associated with the inner membrane of mitochondria. This enzyme complex functions in the catabolism of branched-chain amino acids. Mutations in *BCKDHB* have been associated with maple syrup urine disease, type 1B, an autosomal recessive inborn error that causes mental and physical retardation and feeding problems (RefSeq, PMID 2022752).
NT5DC1	5'-nucleotidase domain containing 1	NT5DC1 belongs to the 5'(3')-deoxyribonucleotidase family (RefSeq).
UNCX	UNC Homeobox	*UNCX* encodes a homeobox transcription factor that is involved in somitogenesis and neurogenesis and is required for the formation of the proximal ribs and the pedicle of the neural arch. *UNCX* also plays a role in controlling the development of connections of hypothalamic neurons to pituitary elements, allowing central neurons to reach the peripheral blood circulation and deliver hormones that control peripheral functions (RefSeq, PMID: 10804168).
MEOX2	Mesenchyme Homeobox 2	*MEOX2* encodes a member of a subfamily of non-clustered, diverged, antennapedia-like homeobox-containing genes. The transcription factor plays a key role in somitogenesis and is required for sclerotome development. Meox2, together with Meox1, functions upstream of Pax genes in the regulation of chondrogenic and myogenic differentiation of paraxial mesoderm (RefSeq, PMID: 12925591).

CREB5	cAMP responsive element binding protein 5	The product of *CREB5* belongs to the CRE (cAMP response element)-binding protein family, which contains zinc-finger and bZIP DNA-binding domains. The encoded protein specifically binds to CRE, and functions as a CRE-dependent trans-activator. *CREB5* showed differential DNA methylation and gene expression between the sexes in both myoblasts and myotubes (RefSeq, PMID: 30646953).
CSMD1	CUB and Sushi multiple domains 1	CSMD1 contains 14 alternating CUB and sushi domains followed by 14 additional tandem sushi domains. It may be an important regulator of complement activation and inflammation in the developing central nervous system. CSMD1 is differentially methylated in a sex- and placental-specific manner, displaying sex-specific differences in placental transcript abundance (PMID: 12906867, 16547280, 29376485).
KIF24	kinesin family member 24	*KIF24* encodes a member of the kinesin superfamily of microtubule-based motor proteins which are involved in the intracellular transport of membranous organelles, protein complexes, and mRNAs. They also play critical roles in mitosis, morphogenesis, and signal transduction. KIF24 binds centriolar coiled coil protein 110 and centrosomal protein 97 and localizes to the mother centriole to regulate ciliogenesis by controlling microtubule polymerization (RefSeq, PMID:21620453).
MIR3974	microRNA 3974	–
FTO	FTO alpha-ketoglutarate dependent dioxygenase	FTO is a nuclear protein of the AlkB related non-haem iron and 2-oxoglutarate-dependent oxygenase superfamily. Studies in mice and humans indicate a role in nervous and cardiovascular systems and a strong association with body mass index, obesity risk, and type 2 diabetes. Mutations in *FTO* result in a severe polymalformation syndrome (RefSeq, PMID: 17434869, 19559399).
TBX1	T-box 1	*TBX1* is a member of a phylogenetically conserved family of genes that share a common DNA-binding domain, the T-box. T-box genes encode transcription factors involved in the regulation of developmental processes. DiGeorge syndrome (DGS)/velocardiofacial syndrome (VCFS), a common congenital disorder characterized by neural-crest-related developmental defects, has been associated with deletions of chromosome 22q11.2, where *TBX1* has been mapped. Studies in mice suggest a major role for *TBX1* in the molecular etiology of DGS/VCFS (RefSeq, PMID:11242110).
**The eQTL transcripts in the previously unreported AIS loci**		
RP11-196G18.22	–	*RP11-196G18.22* is an onco-lncRNA (PMID: 28389669).
RP5-1139B12.3	–	*RP5-1139B12.3* is an RNA gene, and is affiliated with the antisense RNA class.
RP5-1139B12.2	–	*RP5-1139B12.2* is an RNA gene, and is affiliated with the antisense RNA class.

OBSCN	obscurin, cytoskeletal calmodulin and titin-interacting RhoGEF	*OBSCN* contains over 80 exons and encodes a protein of approximately 720 kDa. The protein belongs to the family of giant sacromeric signaling proteins that includes titin and nebulin, and may have a role in the organization of myofibrils during assembly and may mediate interactions between the sarcoplasmic reticulum and myofibrils. Mutations in *OBSCN* may result in the development of a dilated cardiomyopathy phenotype via haploinsufficiency (RefSeq, PMID: 26406308).
WNT9A	Wnt family member 9A	*WNT9A* is a member of the *WNT* family. *WNT* family consists of structurally related genes that encode secreted signaling proteins. These proteins have been implicated in oncogenesis and in several developmental processes, including regulation of cell fate and patterning during embryogenesis. *WNT9A* is clustered with another family member, WNT3A, in the chromosome 1q42 region (RefSeq).
C1orf145	chromosome 1 open reading frame 145	*C1orf145* (*OBSCN-AS1*) is an RNA Gene, and is affiliated with the non-coding RNA class (GeneCards).
FRK	fyn related Src family tyrosine kinase	The protein encoded by *FRK* belongs to the TYR family of protein kinases. This tyrosine kinase is a nuclear protein and may function during G1 and S phase of the cell cycle and suppress growth (RefSeq).
TSPYL4	TSPY like 4	*TSPYL4* is a Protein Coding gene. Diseases associated with *TSPYL4* include Epileptic Encephalopathy, Early Infantile, 6 (GeneCards).
DSE	dermatan sulfate epimerase	The protein encoded by *DSE* is a tumor-rejection antigen. It is localized to the endoplasmic reticulum and functions to convert D-glucuronic acid to L-iduronic acid during the biosynthesis of dermatan sulfate. This antigen possesses tumor epitopes capable of inducing HLA-A24-restricted and tumor-specific cytotoxic T lymphocytes in cancer patients and may be useful for specific immunotherapy. Mutations in *DSE* cause inmusculocontractural Ehlers-Danlos syndrome (RefSeq, PMID: 23704329, 25703627).
FAM26E	family with sequence similarity 26 member E	*FAM26E* is a protein coding gene. An important paralog of *FAM26E* is *FAM26F* (GeneCards).
TPI1P3	triosephosphate isomerase 1 pseudogene 3	*TPI1P3* is a pseudogene, and is affiliated with the antisense RNA class (GeneCards).
FAM26F	family with sequence similarity 26 member F	*FAM26F* is a protein coding gene. An important paralog of *FAM26F* is *FAM26E*. *FAM26F* is significantly enriched in a subset of pediatric AML patients (GeneCards, PMID: 30538250).
RP11-606D9.1	–	*RP11-606D9.1* is an RNA gene, and is affiliated with the lncRNA class.
C22orf29	chromosome 22 open reading frame 29	*C22orf29* (also called Retrotransposon Gag Like 10: *RTL10*) encodes a BH3-only protein (BOP) which could induce apoptosis in a BH3 domain-dependent manner. Diseases associated with *RTL10* include Hemophagocytic Lymphohistiocytosis, Familial, 3. The direct interaction network of Bcl-2 family members may play a key role in modulation RTL10/BOP intrinsic apoptotic signaling activity (GeneCards, PMID: 23055042).

GNB1L	G protein subunit beta 1 like	*GNB1L* encodes a G-protein beta-subunit-like polypeptide which is a member of the WD repeat protein family. Members of this family are involved in a variety of cellular processes, including cell cycle progression, signal transduction, apoptosis, and gene regulation. GNB1L protein contains 6 WD repeats and is highly expressed in the heart. The gene maps to the region on chromosome 22q11, which is deleted in DiGeorge syndrome, trisomic in derivative 22 syndrome and tetrasomic in cat-eye syndrome. *GNB1L* may contribute to the etiology of those disorders
**The nearest genes in the female AIS loci**		
UHMK1	U2AF homology motif kinase 1	*UHMK1* encodes a serine/threonine protein kinase that promotes cell cycle progression through G1 by phosphorylation of the cyclin-dependent kinase inhibitor 1B, which causes nuclear export and degradation. The encoded protein is also thought to function in the adult nervous system and the gene has been associated with schizophrenia. *UHMK1* is also associated with bone mineral density in East Asians and plays a role in osteoblasts and osteoclasts regulation (RefSeq, PMID: 27424934).
SEC16B	SEC16 homolog B, endoplasmic reticulum export factor	*SEC16B* is a mammalian homolog of S. cerevisiae Sec16 that is required for organization of transitional endoplasmic reticulum sites and protein export. *SEC16B* has been associated with adult and childhood body mass index and age at menarche (RefSeq, PMID: 26604143, 21102462).
BOC	BOC cell adhesion associated, oncogene regulated	The protein encoded by *BOC* is a member of the immunoglobulin/fibronectin type III repeat family. It is a component of a cell-surface receptor complex that mediates cell-cell interactions between muscle precursor cells, and promotes myogenic differentiation. *Boc* encodes a SHH coreceptor and is a silent Holoprosencephaly (HPE) modifier gene in mice. Mutations in BOC have been identified in HPE patients (RefSeq, PMID: 28677295).
**The eQTL transcripts in the female AIS loci**		
WDR52	WD repeat-containing protein 52	WDR52, also known as cilia and flagella associated protein 44 (CFAP44), is required for sperm flagella formation and normal sperm motility. Mutations in *CFAP44* are responsible for multiple morphological abnormalities of the flagella syndrome (RefSeq, PMID: 29449551).
WDR52-AS1	WDR52 antisense RNA 1	*WDR52-AS1* (also called CFAP44-AS1) is an RNA Gene, and is affiliated with the non-coding RNA class.

Supplementary Table 8 Numbers of variants in three batches in the analyses

**Study**	**Chr.**	**On array**	**Passing QC before imputation**	**Passing QC after imputation**	**Common across batches**
GWAS1	Autosomes	571,613	387,641	9,272,622	9,059,064
	Chromosome X	15,010	8,807	274,301	263,452
GWAS2	Autosomes	937,171	472,203	9,312,384	9,059,064
	Chromosome X	22,289	9,873	281,328	263,452
GWAS3	Autosomes	943,865	412,149	9,295,288	9,059,064
	Chromosome X	22,496	8,492	279,040	263,452

Chr., chromosome; QC, quality control.

Supplementary Table 9 Statistical power to obtain GWAS significance in the current study

	MAF	OR						
		1.05	1.1	1.2	1.3	1.4	1.5	2
**All**	0.001	7.04E-08	1.41E-07	0.000001	2.22E-06	7.05E-06	1.98E-05	0.000924
	0.005	1.76E-07	9.78E-07	1.89E-05	0.000209	0.001458	0.006991	0.406620
	0.01	3.69E-07	3.85E-06	0.000176	0.003076	0.024959	0.109510	0.971467
	0.03	2.17E-06	0.000080	0.012767	0.210680	0.701936	0.962665	1
	0.05	6.60E-06	0.000467	0.089965	0.685679	0.985603	0.999923	1
	0.1	3.99E-05	0.006133	0.580306	0.996634	1	1	1
	0.2	0.000287	0.063655	0.981107	1	1	1	1
	0.3	0.000811	0.169544	0.999217	1	1	1	1
	0.4	0.001377	0.258231	0.999910	1	1	1	1
	0.5	0.001619	0.290120	0.999956	1	1	1	1
**Female**	0.001	6.78E-08	1.28E-07	5.06E-07	1.76E-06	5.34E-06	1.45E-05	0.000629
	0.005	1.57E-07	7.93E-07	1.35E-05	0.000138	0.000928	0.004429	0.313286
	0.01	3.15E-07	2.91E-06	0.000115	0.001906	0.015673	0.072819	0.939141
	0.03	1.68E-06	5.28E-05	0.007702	0.142493	0.578534	0.917884	1
	0.05	4.82E-06	0.000290	0.056598	0.556826	0.960705	0.999479	1
	0.1	2.68E-05	0.003621	0.446321	0.987259	0.999996	1	1
	0.2	0.000179	0.038492	0.947252	0.999999	1	1	1
	0.3	0.000489	0.107939	0.995704	1	1	1	1
	0.4	0.000817	0.170439	0.999222	1	1	1	1
	0.5	0.000955	0.193375	0.999550	1	1	1	1
**Male**	0.001	5.12E-08	5.48E-08	6.83E-08	0.000000	1.21E-07	1.61E-07	5.68E-07
	0.005	5.63E-08	7.53E-08	0.000000	3.42E-07	6.89E-07	1.31E-06	1.68E-05
	0.01	6.28E-08	0.000000	3.24E-07	9.33E-07	2.41E-06	5.67E-06	0.000152
	0.03	9.01E-08	2.52E-07	1.76E-06	9.38E-06	0.000040	0.000141	0.010784
	0.05	1.20E-07	4.59E-07	5.11E-06	3.85E-05	0.000210	0.000884	0.077350
	0.1	2.02E-07	1.27E-06	2.89E-05	0.000349	0.002534	0.012151	0.536325
	0.2	3.88E-07	4.18E-06	0.000196	0.003428	0.027373	0.117469	0.973370
	0.3	5.65E-07	8.12E-06	0.000542	0.010602	0.079567	0.284782	0.998659
	0.4	6.92E-07	1.16E-05	0.000912	0.018406	0.129206	0.407667	0.999828
	0.5	7.38E-07	1.29E-05	0.001072	0.021729	0.148621	0.449114	0.999915

OR:odds ratio, MAF:minor allele frequency.

Supplementary Note 1

Statistical power of the current study

As shown in Supplementary Table 9, the current study has power to identify variants with low allele frequency (~0.005), if their effect sizes are large (power of 0.4 to identify GWAS significant signal of a variant with OR 2 and MAF 0.005).

Conditional analyses with the use of GCTA-COJO

We also conducted conditional analyses using GCTA-COJO[1] which enable us to conduct conditional analyses without individual genotypes. As a result, we obtained quite similar results (Supplementary Table 3) to those in our conventional conditional analyses. GCTA-COJO resulted in six signals exceeding locus-wide significant level ($p = 5 \times 10^{-6}$). Four out of the six variants (top variants as secondary signals) were also detected by the conventional analyses. One of the two variants not reported in the conventional analyses was explained by different secondary top variants in the same locus with very similar associations (chromosome 7p21.2). The other signal (rs78223574) demonstrated an association slightly below 5×10^{-6} in the conventional analyses (5.6×10^{-6}) and was not reported. We also

noted that one of the secondary signals exceeded GWAS significance in GCTA-COJO (rs13290451, p = 1.6 x 10^{-8} and 8.5 x 10^{-8} in GCTA-COJO and conventional analyses, respectively).

Supplementary Reference

1. Yang, J. *et al*. Conditional and joint multiple-SNP analysis of GWAS summary statistics identifies additional variants influencing complex traits. *Nat. Genet.* **44**, 369-375 (2012).
