# Supplementary material for: Genome-wide association study identifies 14 previously unreported susceptibility loci for adolescent idiopathic scoliosis in Japanese
Source: Nat Commun. 2019 Aug 15;10:3685. doi: 10.1038/s41467-019-11596-w (PMC6695451; doi:10.1038/s41467-019-11596-w)
Supplement: Supplementary file 9 — Reporting Summary [file 41467_2019_11596_MOESM9_ESM.pdf]

nature research

Corresponding author(s): Shiro Ikegawa, Kota Watanabe, and Chikashi Terao

Last updated by author(s): Jul 16, 2019

Reporting Summary

Nature Research wishes to improve the reproducibility of the work that we publish. This form provides structure for consistency and transparency in reporting. For further information on Nature Research policies, see Authors & Referees and the Editorial Policy Checklist.

Statistics

For all statistical analyses, confirm that the following items are present in the figure legend, table legend, main text, or Methods section.

n/a	Confirmed	
☐	☒	The exact sample size (*n*) for each experimental group/condition, given as a discrete number and unit of measurement
☐	☒	A statement on whether measurements were taken from distinct samples or whether the same sample was measured repeatedly
☐	☒	The statistical test(s) used AND whether they are one- or two-sided<br>*Only common tests should be described solely by name; describe more complex techniques in the Methods section.*
☐	☒	A description of all covariates tested
☐	☒	A description of any assumptions or corrections, such as tests of normality and adjustment for multiple comparisons
☐	☒	A full description of the statistical parameters including central tendency (e.g. means) or other basic estimates (e.g. regression coefficient) AND variation (e.g. standard deviation) or associated estimates of uncertainty (e.g. confidence intervals)
☐	☒	For null hypothesis testing, the test statistic (e.g. *F*, *t*, *r*) with confidence intervals, effect sizes, degrees of freedom and *P* value noted<br>*Give P values as exact values whenever suitable.*
☒	☐	For Bayesian analysis, information on the choice of priors and Markov chain Monte Carlo settings
☒	☐	For hierarchical and complex designs, identification of the appropriate level for tests and full reporting of outcomes
☐	☒	Estimates of effect sizes (e.g. Cohen's *d*, Pearson's *r*), indicating how they were calculated

Our web collection on statistics for biologists contains articles on many of the points above.

Software and code

Policy information about availability of computer code

Data collection	We used publicly available software for data collection. The software used is clearly shown in the method section of our manuscript.
Data analysis	We used publicly available software for data analysis. The software used is clearly shown in the method section of our manuscript.

For manuscripts utilizing custom algorithms or software that are central to the research but not yet described in published literature, software must be made available to editors/reviewers. We strongly encourage code deposition in a community repository (e.g. GitHub). See the Nature Research guidelines for submitting code & software for further information.

Data

Policy information about availability of data

All manuscripts must include a data availability statement. This statement should provide the following information, where applicable:
- Accession codes, unique identifiers, or web links for publicly available datasets
- A list of figures that have associated raw data
- A description of any restrictions on data availability

GWAS summary statistics of AIS will be publiclly available at our website (JENGER, http://jenger.riken.jp/en/)

Field-specific reporting

Please select the one below that is the best fit for your research. If you are not sure, read the appropriate sections before making your selection.

☒ Life sciences ☐ Behavioural & social sciences ☐ Ecological, evolutionary & environmental sciences

For a reference copy of the document with all sections, see nature.com/documents/nr-reporting-summary-flat.pdf

Life sciences study design

All studies must disclose on these points even when the disclosure is negative.

Sample size	We aimed to create the largest possible sample size in the Japanese population for the meta-analysis to gain the statistical power.
Data exclusions	We excluded the samples and variants based on the standard quality control procedure in GWAS. Detailed information on quality controls were sufficiently described in the method section of our manuscript.
Replication	Association study was conducted in three different sets of Japanese subjects and the results were combined using fixed-effects meta-analysis. We checked the directional consistency for the significant loci. Because our participating cohorts are the largest cohorts in Japan, there are no Japanese with enough sample sizes.
Randomization	Since we conducted case-control genetic study, we did not randomly allocated subjects into groups.
Blinding	Since we conducted case-control genetic study, blinding is not relevant to our study.

Reporting for specific materials, systems and methods

We require information from authors about some types of materials, experimental systems and methods used in many studies. Here, indicate whether each material, system or method listed is relevant to your study. If you are not sure if a list item applies to your research, read the appropriate section before selecting a response.

Materials & experimental systems

n/a	Involved in the study	
☐	☒	Antibodies
☐	☒	Eukaryotic cell lines
☒	☐	Palaeontology
☒	☐	Animals and other organisms
☐	☒	Human research participants
☒	☐	Clinical data

Methods

n/a	Involved in the study	
☒	☐	ChIP-seq
☒	☐	Flow cytometry
☒	☐	MRI-based neuroimaging

Antibodies

Antibodies used	The HNF-3β (FOXA2) antibody (Santa Cruz, sc-374376x) was used in the gel super-shift assay.
Validation	The antibody used was previously validated by the manufacturer on Western blotting in several cell lines.

Eukaryotic cell lines

Policy information about cell lines

Cell line source(s)	MCF-7 cells was obtained from American Type Tissue Culture (ATCC). OUMS-27 cells was obtained from JCRB Cell Bank.
Authentication	MCF-7 cells and OUMS-27 cells were authenticated by STR analysis.
Mycoplasma contamination	MCF-7 and OUMS-27 cells were negative for mycoplasma contamination.
Commonly misidentified lines (See ICLAC register)	MCF-7 and OUMS-27 cells were confirm the authentication by STR analysis.

Human research participants

Policy information about studies involving human research participants

Population characteristics	All the subjects were Japanese. The number of subjects in the meta analysis were 79,211 and the number of subjects in the female meta-analysis were 38,683.
Recruitment	The case subjects were recruited from collaborating hospitals and diagnosed by expert scoliosis surgeons. The control subjects were recruited from the BioBank Japan project. Detailed information for recruitment was sufficiently described in our manuscript.
Ethics oversight	The ethical committees at all collaborating institutions and RIKEN approved the study.

Note that full information on the approval of the study protocol must also be provided in the manuscript.
