# Supplementary material for: Genome-wide association study identifies 14 previously unreported susceptibility loci for adolescent idiopathic scoliosis in Japanese
Source: Nat Commun. 2019 Aug 15;10:3685. doi: 10.1038/s41467-019-11596-w (PMC6695451; doi:10.1038/s41467-019-11596-w)
Supplement: Supplementary file 10 — Description of Additional Supplementary Files [file 41467_2019_11596_MOESM10_ESM.pdf]

Title: Supplementary Data 1.
Description: Association of the genome-wide significant loci.

Title: Supplementary Data 2.
Description: Heritability enrichment of the 220 cell types.

Title: Supplementary Data 3.
Description: Pathway analysis of the AIS GWAS with PASCAL.

Title: Supplementary Data 4.
Description: Genetic correlation between AIS and the 67 traits.

Title: Supplementary Data 5.
Description: Association of the genome-wide significant loci for female AIS.

Title: Supplementary Data 6.
Description: Functional annotation of SNPs correlated with previously unreported AIS signals ($r^2 > 0.8$).

Title: Supplementary Data 7.
Description: Functional annotation of SNPs correlated with female AIS signals ($r^2 > 0.8$).
